# iPNHOT: a knowledge-based approach for identifying protein-nucleic acid interaction hot spots

**DOI:** 10.1186/s12859-020-03636-w

**Published:** 2020-07-06

**Authors:** Xiaolei Zhu, Ling Liu, Jingjing He, Ting Fang, Yi Xiong, Julie C. Mitchell

**Affiliations:** 1 School of Sciences, Anhui Agricultural University, Hefei, Anhui China; 2 School of Life Sciences, Anhui University, Hefei, Anhui China; 3 School of Life Sciences and Biotechnology, Shanghai Jiao Tong University, Shanghai, China; 4 Biosciences Division, Oak Ridge National Laboratory, Oak Ridge, TN USA

**Keywords:** Protein-nucleic acid interaction, Hot spots, Feature selection, Electrostatic potential, Support vector machine

## Abstract

**Background:**

The interaction between proteins and nucleic acids plays pivotal roles in various biological processes such as transcription, translation, and gene regulation. Hot spots are a small set of residues that contribute most to the binding affinity of a protein-nucleic acid interaction. Compared to the extensive studies of the hot spots on protein-protein interfaces, the hot spot residues within protein-nucleic acids interfaces remain less well-studied, in part because mutagenesis data for protein-nucleic acids interaction are not as abundant as that for protein-protein interactions.

**Results:**

In this study, we built a new computational model, iPNHOT, to effectively predict hot spot residues on protein-nucleic acids interfaces. One training data set and an independent test set were collected from dbAMEPNI and some recent literature, respectively. To build our model, we generated 97 different sequential and structural features and used a two-step strategy to select the relevant features. The final model was built based only on 7 features using a support vector machine (SVM). The features include two unique features such as ∆SASsa^1/2^ and esp3, which are newly proposed in this study. Based on the cross validation results, our model gave F1 score and AUROC as 0.725 and 0.807 on the subset collected from ProNIT, respectively, compared to 0.407 and 0.670 of mCSM-NA, a state-of-the art model to predict the thermodynamic effects of protein-nucleic acid interaction. The iPNHOT model was further tested on the independent test set, which showed that our model outperformed other methods.

**Conclusion:**

In this study, by collecting data from a recently published database dbAMEPNI, we proposed a new model, iPNHOT, to predict hotspots on both protein-DNA and protein-RNA interfaces. The results show that our model outperforms the existing state-of-art models. Our model is available for users through a webserver: http://zhulab.ahu.edu.cn/iPNHOT/.

## Background

The interaction of proteins with nucleic acids is essential in many different cellular processes, such as translation, RNA-metabolism, gene regulation, DNA replication and repair, and so on [1, 2]. The understanding of the interaction between proteins and nucleic acids sheds light on designing new functions and regulating cellular behaviors.

Mutagenesis studies on protein-protein and protein-nucleic acid interfaces provide important clues in exploring the drivers of the interactions [3–5]. It has been shown that the mutation of a few interface residues to alanine can dramatically decrease the binding affinity [6, 7]. Those residues are called hotspots (HS) [8], often defined as a residue whose mutation to alanine generates a binding free energy difference over 2 kcal/mol [9].

While hotspots on protein-protein interfaces have been extensively studied by both experimental and computational methods [6, 9–16], the hotspots on protein-nucleic acid interfaces are not as comprehensively investigated. Possibly, the inherent characteristics, such as electrostatic and hydration of the protein-nucleic acid interfaces, make it difficult to characterize the energetics of mutations. In addition, very few of the energetic data about the residues on protein-nucleic acid interfaces were collected in the past decades, which make the development of computational methods at a slow pace.

Some protein-nucleic acid alanine mutagenesis data from the literature were collected in the ProNIT database [17]. Based on these data, several computational methods had been developed to predict the effect of the mutagenesis or hot spots on protein-nucleic acid interfaces [11, 18–23]. Munteanu et al. developed a model by the combination of an SVM (support vector machine) with a genetic algorithm (GA) as the wrapper for feature selection to predict the hot spots on protein-nucleic acid interfaces based on solvent accessible surface area and residue conservation [11]. Pires et al. used the concept of graph-based signatures to predict the effects of the mutations on protein-nucleic acids interfaces [23]. They built a model called mCSM-NA that can quantitatively predict the effects of the mutations in protein coding regions on nucleic acid binding affinities. These are two methods that can predict hot spots or mutation effect on both protein-RNA and protein-DNA interfaces. Note that mCSM-NA provided different sub-models according to different nucleic acid types. As for protein-RNA interfaces, Barik et al. developed a method, HotSPRing, to predict the hot spots at protein-RNA recognition sites [18]. The model was built by using random forests based on structural and physicochemical features. Recently, Pan et al. developed a method, PrabHot, for predicting hot spots on protein-RNA interfaces by collecting data from ProNIT and literature [24]. On the other hand, for protein-DNA interfaces, Ramos et al. developed a computational alanine scanning mutagenesis methodology to predict the hot spots on protein-DNA interfaces [19]. Peng et al. developed a webserver, SAMPDI (http://compbio.clemson.edu/SAMPDI), which can predict mutation effect on protein-DNA interfaces based on modified MM/PBSA approach [20]. Similarly, Zhang et al. developed model, PremPDI, to predict the mutation effects on protein-DNA interfaces by using molecular mechanics force fields and fast side-chain optimization algorithms [21]. More recently, Zhang et al. developed a feature based model, PrPDH, to predict the hotspots on protein-DNA interfaces [22]. Note that the computational alanine scanning method, SAMPDI and PremPDI are all based on molecular mechanics force fields, so these methods are more time-consuming compared machine-learning based methods.

Although a few computational methods have been developed to predict the mutation effects on protein-nucleic acid interfaces, the data points used in these methods are limited. For example, Pires et al. collected 331 single-point mutations to build mCSM-NA and 79 mutations for testing [23]. Note that their datasets contain all kinds of mutations not just alanine mutation. Barik et al. collected 80 alanine mutagenesis data to build HotSPRing for predicting hotspot on protein-RNA interfaces [18]. Munteanu et al. collected 177 mutations from ProNIT to build their hotspot prediction model [11]. Peng et al. collected 105 all kinds of mutation to build their protein-DNA binding free energy change prediction model [20]. For PrabHot, Pan et al. collected totally 209 mutagenesis data to build and test their model for predicting hotspot on protein-RNA interfaces [24]. For the more recently published model, PrPDH, Zhang et al. collected totally 214 mutagenesis data to build and test their model for predicting hotspot on protein-DNA interfaces [22]. Thus, the generalization of those models could not be well validated due to the limited number of data points.

Because of the limited sample sizes for only protein-DNA or protein-RNA interfaces, in this study, alanine mutagenesis data on both protein-DNA and protein-RNA interfaces were collected from a comprehensive database dbAMEPNI [25] and other published literature. Then, seven kinds of sequential or structural features were generated for the interface residues. Based on the features, we were able to develop a knowledge-based model to predict the HS on both protein-DNA and protein-RNA interfaces by using a two-step feature selection strategy.

## Methods

### Benchmark datasets

#### Training dataset

The training dataset comes from dbAMEPNI database [25] which was built in our group. The database contains alanine mutagenic effects data from ProNIT database [17] and our curated data collected from literature between 2011 and 2017. Note that the data collected from literature between 2017 and 2018 has been used as a part of the independent test set.

Firstly, we identified 335 interface residues from dbAMEPNI database by defining the interface residue as a residue whose buried solvent accessible surface area is larger than 0.0 when binding. Then, we detected redundancy among homologous proteins using the PISCES server [26] with a sequence identity cutoff set to 25%. When the sequence identity between two proteins is higher than 25%, we aligned the 3D structures of the two proteins in PyMol and then observed the binding sites of the two proteins. If the environment of the binding sites is different between the two proteins, we kept the corresponding binding sites. Finally, we obtained a dataset containing 293 interface residues, which come from 105 protein-nucleic acid complexes that consist of 74 protein-DNA complexes, 30 protein-RNA complexes and 1 protein-RNA/DNA complexes. The complexes and the 293 interface residues are listed in Table S1 of Additional file 1 and S2 of the Additional file 2, respectively. According to Table S2, 102 interface residues are common to the data in ProNIT. By using ∆∆G = 2.0 kcal/mol as cutoff, 86 of the 293 interface residues are defined as hot spot residues and the remaining 207 residues are defined as non-hot spot residues.

#### Independent test set

The independent test set comes from four different sources: (i) The independent test set in Pires et al.’s work which comes from Barik et al.’s paper [18]. In their paper, they collected 80 alanine mutations from 14 protein-RNA complexes. Note that these alanine mutations were also included in the dbAMEPNI database later. After checking these protein-RNA complexes, we found one complex (PDBID: 2Y8W) appeared in our training dataset, so we removed this complex. The other complex 2XS2 was also removed because the only one corresponding residue is not on the protein-RNA interface. (ii) The independent test set reported in Pan et al.’s work [24]. In their work, they collected 58 mutations as their independent test set. After carefully checking the 58 mutations, we found several problems of the dataset: (1) It includes 13 non-alanine mutations; (2) The multiple mutants were wrongly considered as single alanine mutants. For example, the mutants of 4JVH are all double mutants, however, they were used as single alanine mutants in their dataset; (3) There is redundancy between 5EN1 and 5HO4. According to these problems, we removed part of the data and kept 33 data points. We have summitted the 33 data points on the PrabHot server, and 23 PrabHot scores (Table S4 in Additional file 2) were available for plotting the receiver operating characteristic (ROC) and precision recall (PRC) curves. (iii) Literature corresponding to the 3D structures of protein-nucleic acid complexes available in PDB database [27] from 2017 to 2018. We identified 51 protein-nucleic acid complexes, and the corresponding references were carefully examined to find the alanine mutation information. From these articles, we obtained 16 alanine mutation data. (iv) The dataset used in Peng et al.’s paper [20]. In their paper, the authors collected 105 missense mutations from 13 proteins, of which 6 proteins (PDB code: 1FOS, 1HCQ, 2MXF, 3UFD, 4ATK and 4RDU) were not overlapped with the data in dbAMEPDI. However, the mutations of 4ATK and 4RDU are not alanine mutation. Thus, we obtained 32 alanine mutation data from their paper.

**Fig. 1:**
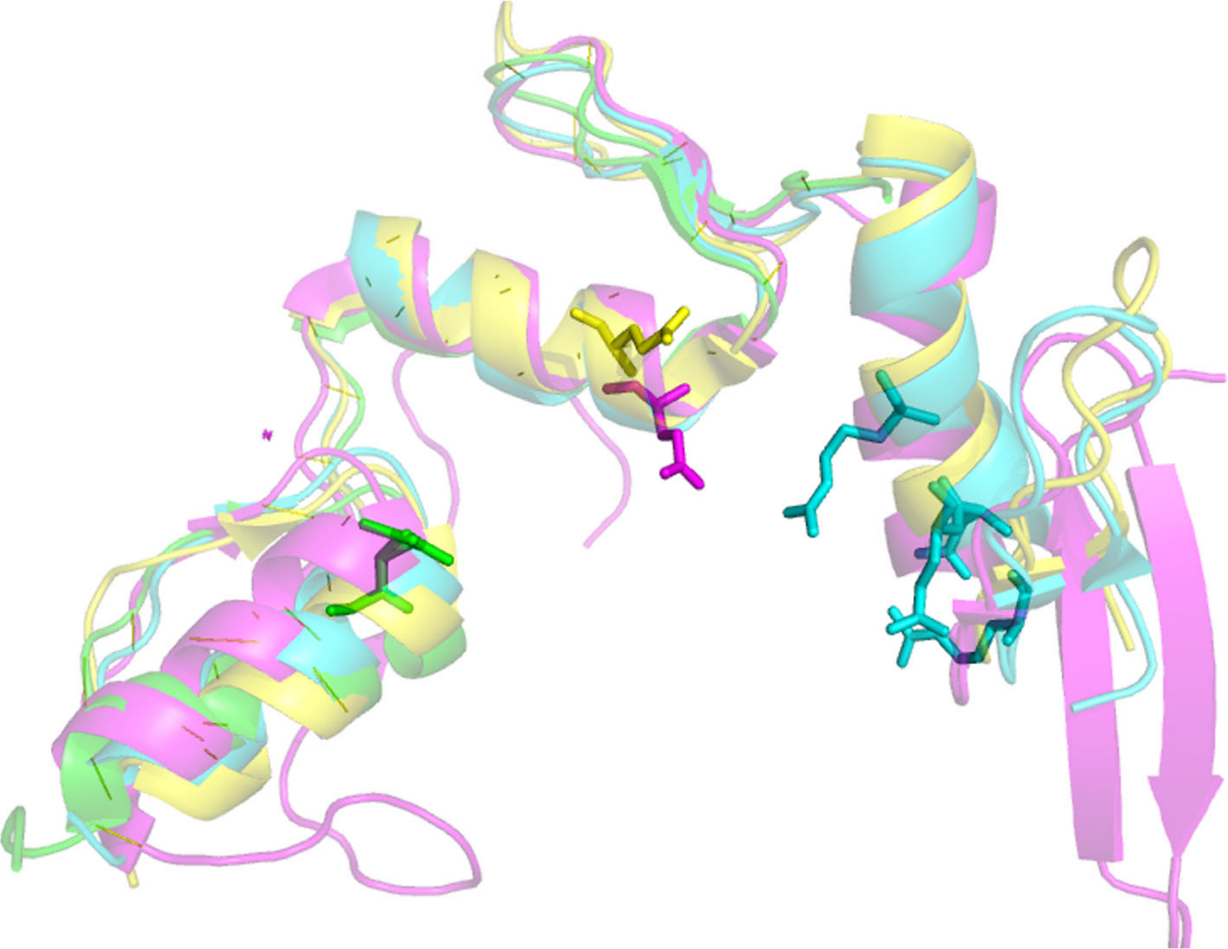
An example shows the different recognition sites between homologous protein pairs. Green:4GZN; Cyan: 1AAY; Yellow: 4M9E; Magenta: 5VMV

For the proteins collected from the four sources mentioned above, we used PISCES server to determine the sequence identity between them and the proteins in the training dataset using the sequence identity cutoff 25%. Similarly, we used PyMol to align the two proteins and observe the binding sites if the sequence identity between the proteins is higher than 25%. Table 1 shows those homologous pairs and their recognition sites on the protein-nucleic acid interfaces. Figure 1 shows an example that indicates how we aligned the structures of homologous pairs and observed their recognition sites. As shown in Table 1, the four protein chains 4GZNC, 1AAYA, 4M9EA and 5VMVA are homologous pairs, Fig. 1 shows clearly that the referred recognition residues are different although the sequence identity between those chains are higher than 25%. In all, we obtained 124 interface residues which come from 32 protein-nucleic acids complexes that consist of 22 protein-RNA complexes, 9 protein-DNA complexes and 1 protein-RNA/DNA complexes (Table S3 in Additional file 1). By using ∆∆G = 2.0 kcal/mol as cutoff, 14 of the 124 interface residues are hotspot residues and 110 of them are non-hot spot residues (Table S4 in Additional file 2).

**Table 1:** Homologous pairs in both training and test dataset with the sequence identity and the recognition sites

Protein1(dataset)^a^	Protein2(dataset)	Sequence identity(%) ^b^	Recognition site1^c^	Recognition site2^c^
4GZNC (train)	1AAYA (train)	33	E182	R118,D120,E121,R124
4GZNC (train)	4M9EA (train)	34	E182	E446
4GZNC (train)	5VMVA (test)	27	E182	E535
5EXHC (train)	3QMGA (train)	34	T80,H81,Q82,K88	Q201,R213,Y216
4ALPA (train)	5UDZA (train)	74	F77	Y140,H148,H162
5EIMA (train)	5DNOA (train)	100	R349,K436,T437,N477	N336,R338
5DFFA (train)	4B5FA (train)	35	R181	N207,R208
4RCJA (train)	4R3IA (train)	29	Y397	R475
3WPCA (train)	5ZLNA(test)	72	W47,F108,W96	F375,F402,Y537
5GXHA (train)	5H1KA (train)	99	F381,E197,Y474	N13,W14,Y15,R33,M357,R359
5U2RA (train)	2BPFA (train)	96	R283	K280,N294
5U2RA	5U8GA (train)	100	R283	M236
5U2RA	1BPXA (test)	100	R283	R283
5U2RA	4X5VA (train)	35	R283	N513
5U2RA	5TWPA (train)	26	R283	W434, H329
5U2RA	4XQ8B (train)	34	R283	Y505
5U2RA	5IIIA (train)	34	R283	E529
5HO4A (test)	2ERRA (train)	28	Q19,F66,E92,D49,F24,H108	H120,F160,F158,F126
5HO4A	2KXNB (test)	28	Q19,F66,E92,D49,F24,H108	I195,T196,P199,S194,R111
5HO4A	4CIOA	32	Q19,F66,E92,D49,F24,H108	N106,Y44,N108
4L5RC (train)	3RN2A (train)	40	N236	K160,R244,K335,R311,K251,K198,K309,K204
4HN5A (train)	1HCQA (test)	45	K442	S15,H18,Y19,E25,K32
4HT8A (train)	3QSUA (train)	31	Y25	K33
4HT8A (train)	4QVCD (train)	100	Y25	N48,N28,K31
3SPDA (train)	3SZQA (train)	98	H138,S142	F65
3OSGA (train)	1MSEC (train)	39	K49,R84,N139,K138,R87,F52,K51	S187
3EQTA (test)	5JBJA (train)	43	E573	H406
1QRVA (train)	1J5NA (train)	38	V32,L97	K53,Y81,N33,R23,R36,Y28,K67,R40,Y88,K60,M29,F48,K78,K22,K85
3OD8A (train)	3ODCA (train)	32	F44,V48	R122,L151,R138,I154
5FD3A (train)	4RKGA (train)	31	Y610,Y536	R526,R543
2I05A (train)	1ECRA (train)	100	R198	Q250,K89
2I05A (train)	4XR0A (train)	100	R198	H144

^a^The first four letters are the PDB code and the fifth letter is the chain ID. The remark in the parentheses is the dataset that the protein-nucleic acids complexes belong to

^b^Homologous pairs are defined using sequence identity cutoff value 25%

^c^The first letter is the residue name in one letter, and the numbers after the letter is the residue sequence number in the protein

### Feature extraction

Due to the special characteristics of the protein-nucleic acid interfaces, we generated 7 different kinds of features to build our model, which were described as below.

#### Physicochemical characteristics of 20 amino acids

As the basic unit that comprises a protein, it is the residues that interact with other molecules. Different properties of the 20 residues have been deposited in AAindex [28], 10 of which were used as features to predict the hotspots on protein-protein interfaces in previous studies [29–32]. The 10 physicochemical properties were considered as the first 10 features as shown in Table S5 (see Additional file 1). The numerical values of the 10 features are shown in Table S6 (see Additional file 1).

#### Depth index and protrusion index

The surface shape complementarity between proteins and nucleic acids is an important factor in protein-nucleic acid binding. The surface geometry of residues in the interface is quantified using features in this study. We used the PSAIA [33] program to calculate the depth index (DI) and protrusion index (PI) for each interface residue. The program calculates several different kinds of depth index and protrusion index including the average values of the entire and side chain of the residue, the maximum and minimum values of the residue’s atoms. We used the first 4 values for each residue in both bound and unbound state as features. More specifically, these features contain the average DI of the entire residue, the average DI of the side chain of the residue, the average PI of the entire residue, and the average PI of the side chain of the residue.

In addition, we calculated the differences of these 4 values of each residue between bound and unbound states by using the following equations:

1 $$ \Delta DIt= DItu- DItb $$

2 $$ \Delta DIs= DIsu- DIsb $$

3 $$ \Delta PIt= PItu- PItb $$

4 $$ \Delta PIs= PIsu- PIsb $$

where, the *DItu* and *DItb* mean the average DIs of the total residue in unbound and bound states, respectively. The *DIsu* and *DIsb* mean the average DIs of the side chain of the residue in unbound state and bound states, respectively. We did the same for PI. Furthermore, we calculated the relative DIs and PIs according to the following equations:

5 $$ relDIt=\Delta DIt/ DItu $$

6 $$ relDIs=\Delta DIs/ DIsu $$

7 $$ relPIt=\Delta PIt/ PItu $$

8 $$ relPIs=\Delta PIs/ PIsu $$

In all, we obtained 16 features related to depth index and protrusion index.

#### Features related to solvent accessible surface area (SASA)

The residue’s solvent accessible surface area has been used as features in previous studies [11, 14, 34–36] in predicting hotspots on protein-protein interfaces. In this study, we used the different representations of SASA as features to build our model. The SASA was calculated by NACCESS program [37], which calculated the SASA of a residue in different scenarios, for example, the absolute SASA and the relative SASA, the SASA of all atoms, side chain atoms, backbone atoms, polar, and nonpolar atoms of the residue. We obtained these SASAs in both bound and unbound states.

In addition, we calculated the buried SASA that is the difference of the SASA between proteins in bound and unbound states. The buried SASA has been thought to correlate with different energy terms such as desolvation energy. In this work, we calculated different kinds of buried absolute SASA and relative SASA mentioned above. Furthermore, we considered different powers of the buried absolute SASA and relative SASA as features. The three powers we tested are 0.5, 1.5, and 2.0.

In all, we obtained 54 features related to SASA. These features can be found in Table S5 (see Additional file 1).

#### Features related to electrostatic potential

Considering the electrostatic characteristics of nucleic acids, the electrostatic potential could be benefit for predicting hotspots on protein-nucleic acid interfaces. In this study, we used the APBS program [38] to calculate the electrostatic potential around the proteins, and the procedure to calculate the electrostatic potential of a residue has been described in our previous study [39]. The description of the 5 features related to electrostatic potential can be found in Table S5.

#### Hydrogen bond features

By using the WHATIF server [40] we obtained the hydrogen bonds [41] on protein-nucleic acid interfaces. The hydrogen bond numbers formed by the entire residue and those of the side chain with nucleic acid were counted as two features.

#### Secondary structure features

A residue’s secondary structure is assigned by the DSSP program [42, 43], which outputs 8 different kinds of secondary structure that include H (α-helix), B (isolated β-bridge), E (extended strand), G (3-helix), I (5-helix), T (hydrogen bonded turn), S (bend), and blank (loops). We re-categorized them into 5 different types by combining B, T, S as turn, and G, I as helix1. Then the 5 different types of secondary structure were represented as binary vectors by using (1, 0, 0, 0, 0) as H, (0, 1, 0, 0, 0) as E, (0, 0, 1, 0, 0) as turn, (0, 0, 0, 1, 0) as helix1, and (0, 0, 0, 0, 1) as loops.

#### Sequence conservation features

Based on our previous works [39, 44], we obtained 5 features from the PSSM file generated by PsiBlast. The first one is the information entropy that represents the conservation of the corresponding sequence position. In addition, we defined two kinds of relative conservation based on the weighted observed percentage of each kind of residues for each sequence position as follows:

9 $$ CNSV\_ REL{1}_{wop}={\hat{P}}_{ra}/{\hat{P}}_A $$

10 $$ CNSV\_ REL{2}_{wop}={\hat{P}}_{rm}/{\hat{P}}_A $$

where, ${\hat{P}}_x={P}_x+1$, *P*_*x*_ is the weighted observed percentage of residue type *x* at the certain sequence position, with the formulas designed to avoid division by 0. *P*_*A*_ is the weighted observed percentage of the residue type “alanine” at the certain sequence position. Label ‘rm’ means the residue type with the maximum percentage, and ‘ra’ means the actual residue type at that sequence position. And ‘wop’ is the abbreviation of ‘weighted observed percentage’. Similarly, we also defined two kinds of relative conservation based on the position specific scores in the position specific scoring matrix (PSSM) as follow:

11 $$ CNSV\_ REL{1}_{pps}={S}_{ra}-{S}_A $$

12 $$ CNSV\_ REL{2}_{pps}={S}_{rm}-{S}_A $$

where, *S*_*x*_ is the position specific score of residue type *x* on the certain sequence position. Labels ‘ra’, ‘rm’, and ‘A’ have the same meaning as above, and ‘pps’ is the abbreviation of ‘position specific score’.

In all, we obtained 97 features in this study, and the z-scores were calculated to standardize all the features.

### Feature selection

Feature selection has become an important step for building machine learning models, especially for high-dimensional applications. By feature selection, redundant and irrelevant features can be removed, and we can also avoid over-fitting, improve model performance and provide faster and more cost-effective models.

Previous study [44] shows that a hybrid two-step feature selection strategy is effective to detect relevant feature subset. In this work, we combined decision tree and sequential forward feature selection as a two-step strategy to determine the relevant feature subset. First, we used a MATLAB function FITCTREE to select a feature subset. FITCTREE conducts the CART decision tree algorithm, which gives the best subset of features to discriminate hotspots and non-hot spot residues. A decision tree is a tree whose internal nodes are tests on input patterns and whose leaf nodes are categories of patterns. Then, we used the sequential forward feature selection (SFS) method to determine the final feature subset.

For comparison, we also used NSGA-II (Non-dominated Sorting Genetic Algorithm II) and Boruta algorithm to select the features. NSGA-II (Non-dominated Sorting Genetic Algorithm II) is a popular method for multiple objective optimization [45]. The Boruta algorithm is a wrapper-base feature selection method, which built using random forest [46].

### Evaluation with SVM

Support vector machine (SVM) has been used to build models for predicting hotspots on protein-protein interfaces in several previous studies [11, 14, 15, 34], due to its low complexity and robust output. In this study, SVMlight [47] and the radial basis function were used to train our models. The two parameters, G and C, were optimized by a grid search with G values from 0 to 2 and C values from 0 to 40. To avoid over-fitting, we used the leave-one-out cross validation to evaluate the models. Then, the model was further validated on an independent test set.

Due to the imbalance of our data set, the overall accuracy is heavily biased by the accuracy of the negative examples. Therefore, we provide several different metrics, sensitivity (SEN), specificity (SPE), accuracy (ACC), precision (PRE), F1 score and Matthew correlation coefficient (MCC) to evaluate the performances of different models. These metrics are defined as follows:

13 $$ SEN= TP/\left( TP+ FN\right) $$

14 $$ SPE= TP/\left( TN+ FP\right) $$

15 $$ ACC=\left( TP+ TN\right)/\left( TP+ FN+ TN+ FP\right) $$

16 $$ PRE= TP/\left( TP+ FP\right) $$

17 $$ F1\; score=2 TP/\left(2 TP+ FN+ FP\right) $$

18 $$ MCC=\frac{TP\times TN- FP\times FN}{\sqrt{\left( TP+ FP\right)\left( TP+ FN\right)\left( TN+ FP\right)\left( TN+ FN\right)}} $$

where, *TP*, *FP*, *TN* and *FN* represent the numbers of true positive (predicted hot spot residues are actual hot spots), false positive (predicted hot spot residues are actual non-hot spots), true negative (predicted non-hot spot residues are actual non-hot spots) and false negative (predicted non-hot spot residues are actual hot spots), respectively. In addition to these 6 parameters, the Areas under the Curve (AUC) of the Receiver Operating Characteristic (ROC) curve and the Precision-Recall curve (PRC) were also used as metrics to evaluate our model. The ROC curve shows the relationship between true positive rate and false positive rate, and the area under the curve of ROC curve (AUROC) indicates how strongly the model separates the positive and negative examples. The PRC curve shows the relationship between precision and recall, and the area under the curve of PRC curve (AUPRC) can also evaluate the model’s performance.

### Statistical analysis to detect the relationship between features and hotspots (Wilcoxon rank sum test)

Statistical analysis is useful to reveal the role of each feature on differentiating hot spots from non-hot spots. Because the normal distribution of our data was not guaranteed, the t-test could not be used to analyze the selected features. Instead, the Wilcoxon Rank Sum test was used in the statistical analysis. The Wilcoxon Rank Sum test is a nonparametric test to assess whether two samples of observations come from the same distribution. The RANKSUM function in MATLAB was used in this statistical analysis.

## Results and discussion

### Composition and position distribution of the residues in the datasets

**Fig. 2:**
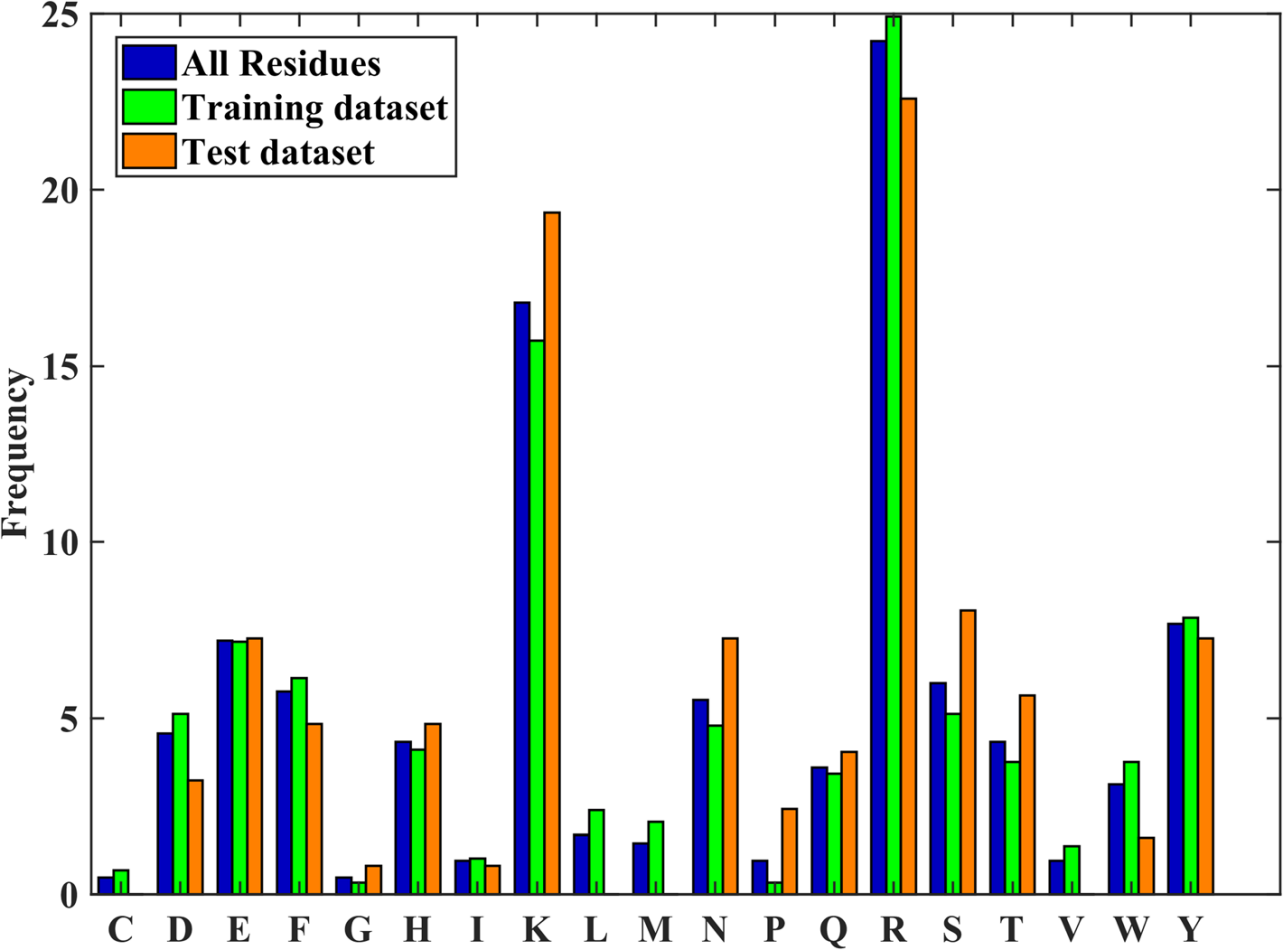
The percentages of the 19 types of residues in our datasets

To give an intuition of the interface residues on protein-nucleic acids interfaces, we analyzed the composition and position distribution of the residues in our datasets. Figure 2 shows the percentages of the 19 types of residues in our training dataset, independent test set and both of the data sets. It is clear that the two positive charged residues, ARG and LYS, have the highest frequencies. This is normal because of the negative charges of phosphate groups of nucleic acids.

**Fig. 3:**
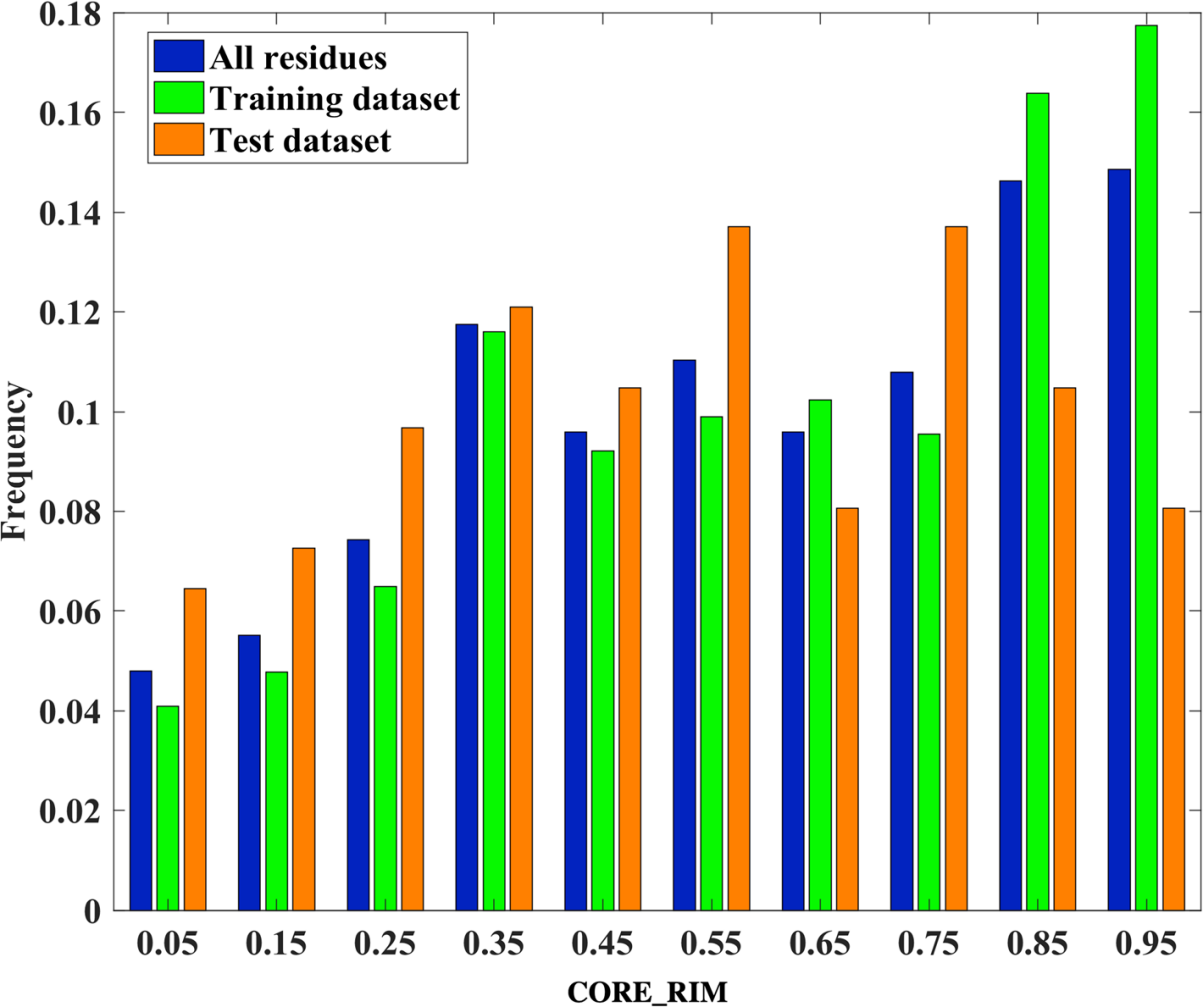
The position distribution of the residues in our datasets by using CORE_RIM values

The position of the interface residues in the datasets were described by a CORE_RIM feature that was proposed in our previous paper [14]. The CORE_RIM value is defined as the (SAStau-SAStab)/SAStau, note that SAStau and SAStab are the feature 29 and 39 in Table S5 in Additional file 1. Figure 3 shows that overall our datasets include both residues on core and rim parts of the interfaces, although the residues in core positions are a little bit more than the residues in rim positions (see the blue bars). In addition, the ratio of core residues in the training dataset is higher than that of the independent test set.

### Feature selection

#### The correlations between the 97 features

**Fig. 4:**
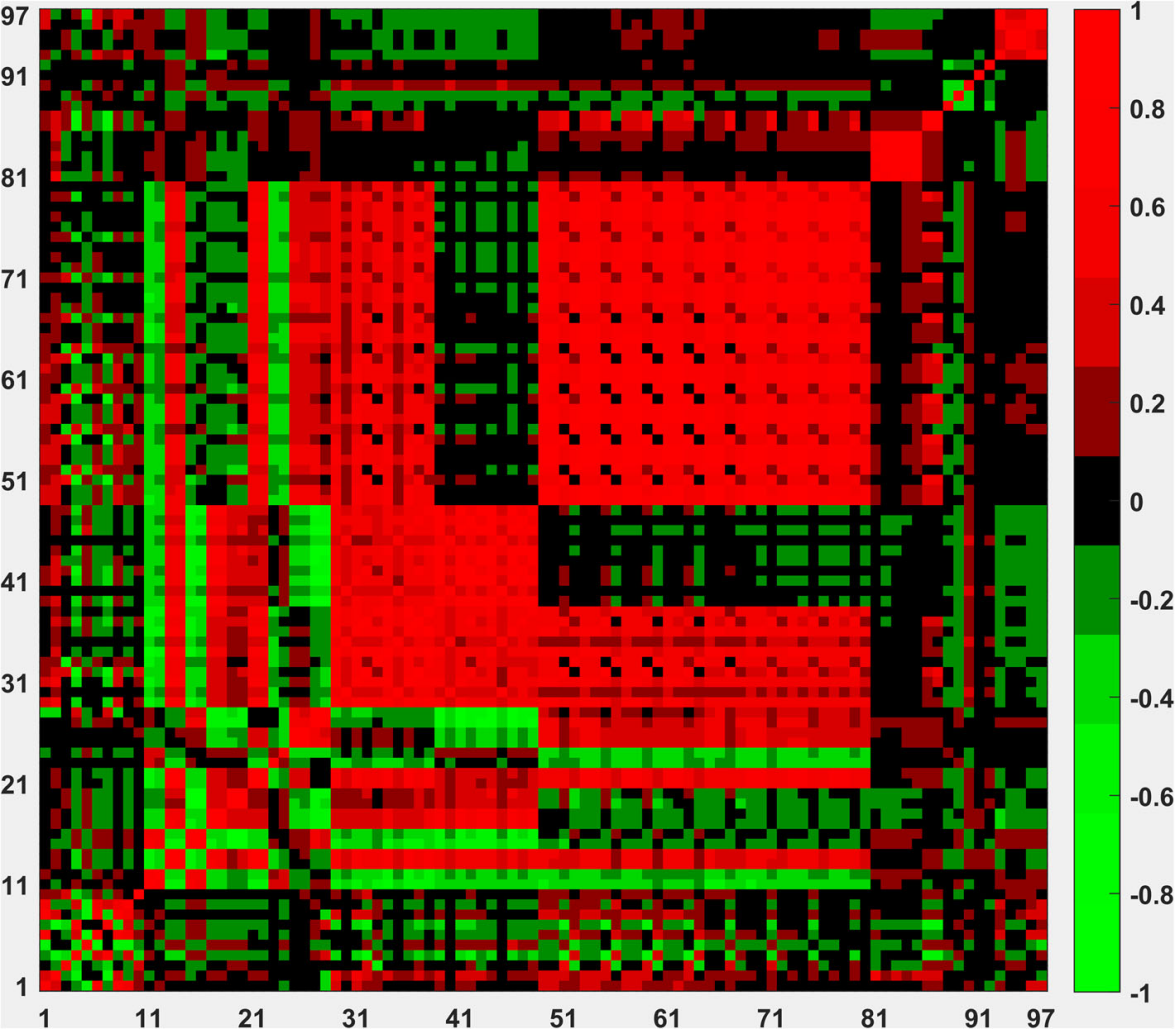
The correlation coefficients between the generated 97 features

In this study, we generated totally 97 features which come from 7 different kinds of structural or sequential properties. The features from each structural or sequential properties may be interdependent and the features from different properties could be also interdependent. We calculated the correlation coefficients between different features. Figure 4 shows the correlation coefficients between different features. It shows that the features from the same structural or sequential properties are easily interdependent, for example, the feature 29–48 are highly correlated because they are all solvent accessible surface area related features and the features 49–80 are also highly correlated because they are based the differences of solvent accessible surface areas between bound and unbound states. The features from different structural or sequential features are generally less interdependent, for example, the correlation between electrostatic potential features (features 81–85) are generally independent to other features.

According to the correlation analysis between different features, a feature selection is necessary to find an optimal feature subset to build our model.

#### Features selected by decision tree

As shown in List 1, the decision tree selected 20 features from 97 original features, which include 3 physicochemical features of amino acids, 7 features related to depth index and protrusion index, 6 features related to solvent accessible surface area, 2 features related to electrostatic potential, 1 feature related to secondary structure, and 1 feature related to conservation.

**List 1.** The features selected by Decision tree. The number in the parenthesis is corresponding to the feature number in Table S5.

**Table:** 

*∆DIs* (20), CNSV (93), *∆*SASsa^1/2^ (50), *∆PIs* (22), Helix (88), *esp*1 (81), *esp*3 (83), SASpau(37), Na (1), *∆DIt* (19), SASbau (33), *∆*SASnr^1/2^ (68), PIsu (14), DIsb (16), SAStau (29), Nphb (3), PItu (13), *∆*SASta^1/2^ (49), Hdrpo (4), DItb (15)	

#### The final feature subset selected by SFS

From the preliminary subset of features selected by decision tree, we further used a sequential forward feature selection (SFS) process to determine the final subset of features as input of the final model. In each round of the SFS process, different feature combinations were used to train models by SVM, and the cross-validation results (F1 score) were used to evaluate these feature combinations. Thus, the contribution of each remaining feature was identified, then the features contribute more were selected. This strategy was also used in Yang et al.’s work [48]. We selected the top three feature combinations in each round for the next round. Table S7 in the Additional file 1 shows the features selected in each round and the corresponding cross validation F1 scores. The results show that the predictive performance is convergent at the 7th round. The best cross-validated F1 score is 0.684. The corresponding feature combination contains 7 features, which are Nphb, PItu, *∆DIs*, SAStau, *∆*SASsa^1/2^, *esp*3, and Helix. Nphb is the number of potential hydrogen bonds of the residue, which means the number of possible hydrogen bonds that a residue can formed with other molecules. PItu is the total protrusion index of the residue in unbound state. *∆DIs* is the difference of the side chain depth indexes between bound and unbound states. SAStau is the total absolute SASA of the residue in the unbound state. *∆*SASsa^1/2^ is the square roots of the differences of the absolute SASAs of residue side chain between unbound and bound states. *esp*3 is the electrostatic potential of the neighbor residues and the target residue. Helix describes if the residue lies in a helix secondary structure.

Based on these 7 features, we built our final model, iPNHOT (identification of protein-nucleic acid interaction hot spots), using SVM. The parameters of G and C for radial basis function used in the final model are 0.1 and 40.0, respectively. The cross-validation results show that our model achieved 0.628, 0.750, 0.684, and 0.829 for recall, precision, F1 score and accuracy, respectively.

**Fig. 5:**
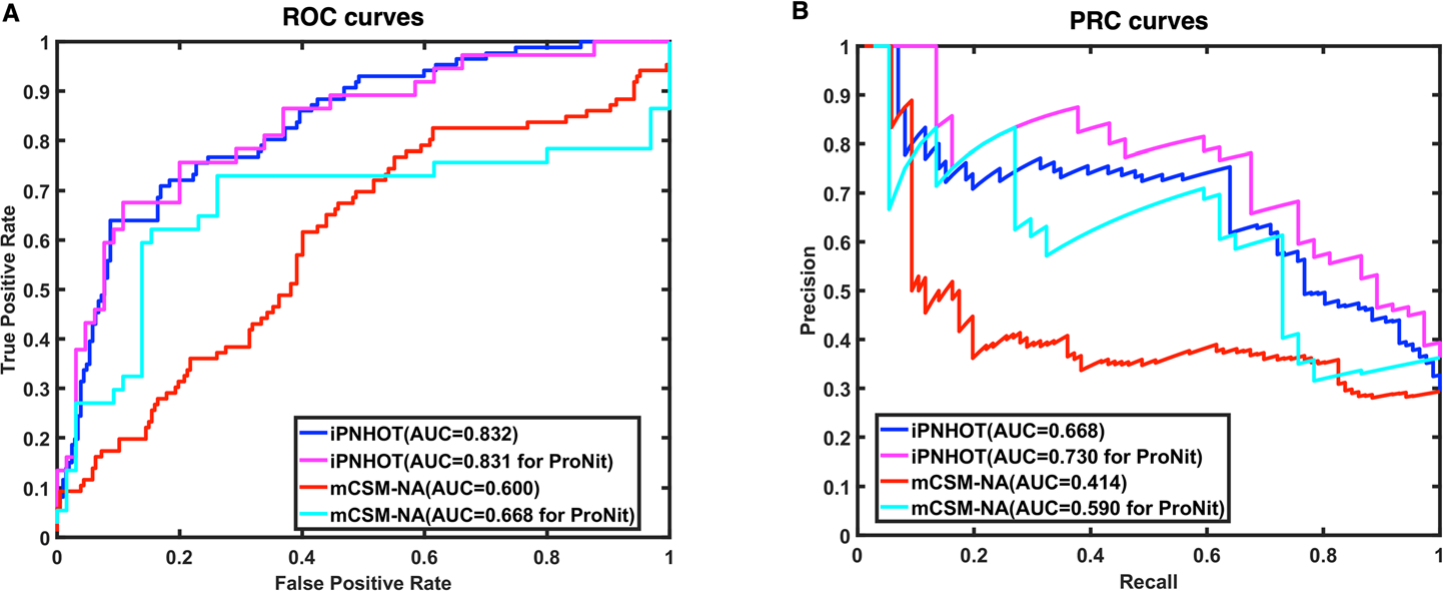
The ROC and PRC curves of the cross-validation results of iPNHOT and the predictive results of mCSM-NA on the training data set. A. ROC curves; B. PRC curves

In addition, we plot the ROC and PRC curves based on the cross-validation results as shown in Fig. 5a and b. The AUROC and AUPRC are 0.832 and 0.668, respectively.

Our previous work [14] in predicting hotspots on protein-protein interfaces showed that differences between the leave one-residue out cross validation and the leave one-protein out cross validation is small. Briefly, the leave one-residue out cross validation is the standard leave one out cross validation in our case, for a sample in our dataset is corresponding to a residue. When we do the leave one-protein our cross validation, the samples belong to a protein were used as the validation set and the samples belong to the other proteins were used to train a model. In this work, we also did a leave one-protein out cross validation based on the final feature subset. The results indicated that the leave one-protein out cross validation achieved the sensitivity, specificity and F1 score of 0.535, 0.894 and 0.597, respectively, which is worse than that of the leave one-residue out (i.e. the standard leave one out) cross validation.

### Models based on all features or the features selected only by decision tree or SFS

To validate the effectiveness of our two-step feature selection process, we also built models based on all 97 features (AFmodel), the 20 features selected only by decision trees (DTmodel), and the features selected only by SFS (SFSmodel), respectively. As shown in Table 2, the AFmodel gives the lowest predictive accuracies compared with other models. The iPNHOT model is superior to DTmodel on all the six evaluation metrics. We inferred that using all features or the 20 features selected by decision tree may have over-fitted the models. In addition, we did the SFS feature selection based on the original 97 features, although it is five more times time-consuming than our two-step feature selection process for each round. Table S8 in the Additional file 1 shows the SFS process, it was convergent at the 9th round. Table 2 shows that the SFSmodel is superior to iPNHOT model on all the six evaluation metrics except specificity, however, the SFSmodel are easily overfitted.

**Table 2:** Cross-validation results of models based on all features and the features selected by only decision tree, only sequential forward selection, and our two-step feature selection process

Models	REC	PRE	SPE	ACC	F1 score	MCC
iPNHOT	0.628	0.750	0.913	0.829	0.684	0.572
DTmodel	0.570	0.681	0.889	0.795	0.620	0.485
SFSmodel	0.709	0.763	0.908	0.850	0.735	0.631
AFmodel	0.442	0.567	0.860	0.737	0.497	0.327

**Fig. 6:**
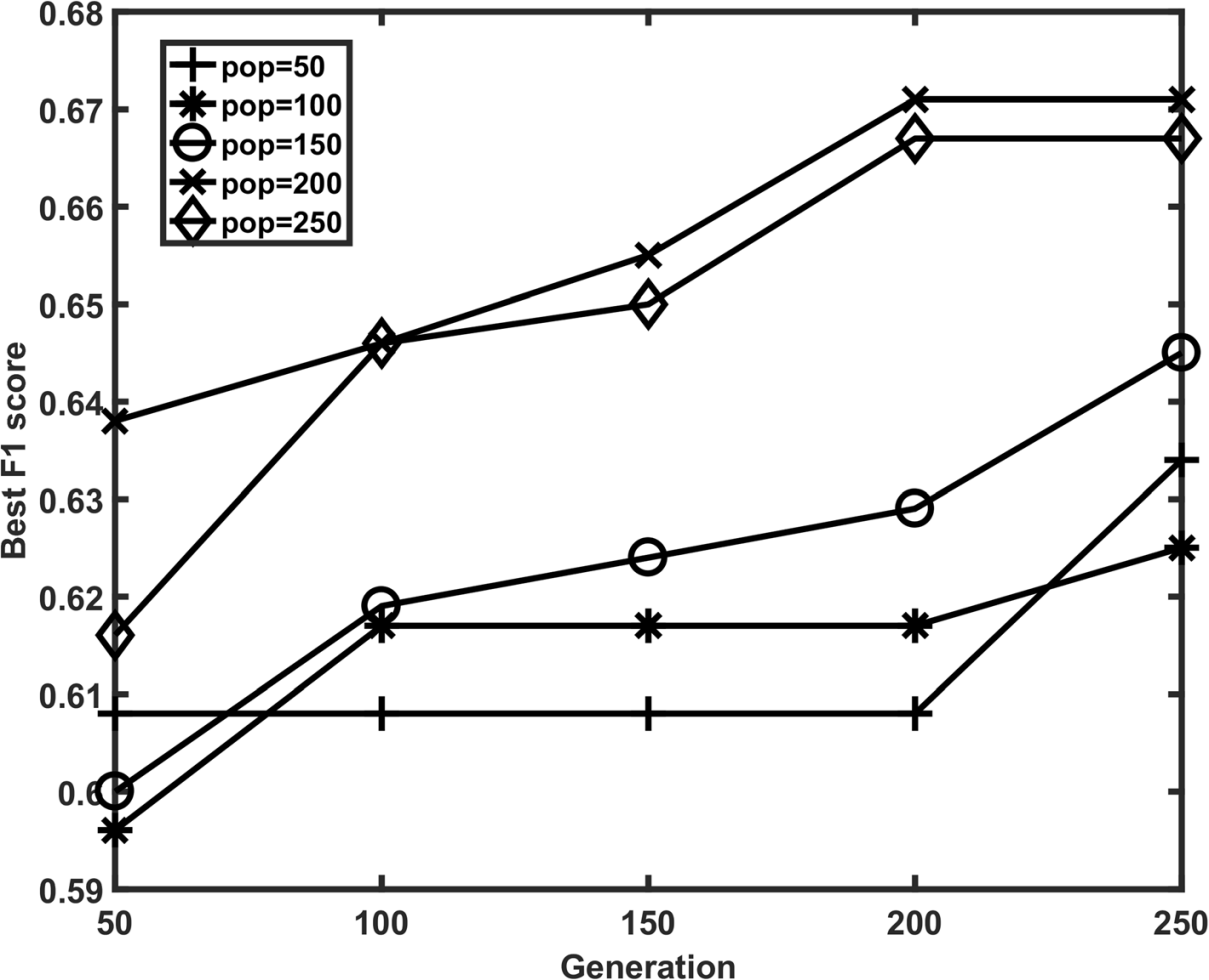
The different F1 scores obtained with different populations and generations of NSGA-II

To further demonstrate the effectiveness of our two-step feature selection strategy, we also combined NSGA-II and SVM to select the relevant feature subset and optimize the G and C parameters of SVM. We tried different populations (50–250) and different generations (50–250) of NSGA, as shown in Fig. 6, the best F1 score is 0.671 which was obtained when population and generation were set to 200 and 200, respectively. In addition, we also built model based the features selected by Boruta algorithm, which selected 16 features. Based on the selected features, we did the cross validation on the training dataset and obtained the best F1 score 0.523. Thus, we show that our two-step feature selection strategy is superior to GA and Boruta algorithm in this study.

### Comparison of different classifiers on the selected 7 features

To evaluate the effectiveness of the SVM learning method in predicting the hot spots within protein-nucleic acid interfaces, we compared the performance of models built by different machine learning algorithms (KNN, naïve Bayesian (NB) and Logistic Regression (LR)) based on the selected 7 features. Table 3 shows that the model built based on SVM (iPNHOT) achieved the highest recall (0.628), the highest precision (0.750), the highest accuracy (0.829), the highest F1 score (0.684), and the highest MCC (0.572) compared with other models. These results indicated that the SVM model outperformed the models built by KNN, naïve Bayesian, logistic regression based on the selected 7 features.

**Table 3:** Cross validation results of different classifiers based on the selected 7 features

Learning algorithms	REC	PRE	SPE	ACC	F1 score	MCC
KNN1	0.570	0.533	0.792	0.727	0.551	0.355
KNN3	0.512	0.595	0.855	0.754	0.550	0.384
KNN5	0.454	0.574	0.860	0.741	0.507	0.338
NB	0.384	0.579	0.884	0.737	0.462	0.308
LR	0.302	0.520	0.884	0.713	0.382	0.226
Random Forest^a^	0.430	0.649	0.903	0.765	0.517	0.384
SVM (iPNHOT)	0.628	0.750	0.913	0.829	0.684	0.572

^a^The Random forest model is based on the all 97 features generated in this study, and the corresponding tree number is 68

In addition, to further evaluate the effectiveness of our feature selection process and the SVM learning method in predicting the hot spots on protein-nucleic acid interfaces, we also compared our iPNHOT model with the random forest model built using all the 97 features. We used all the 97 features because random forest classifier is an ensemble learning method and the diversity of trees is important for the algorithm. One of the important steps of the random forest algorithm is to select a feature subset randomly, then to determine an optimal feature from the feature subset to divide the examples. Thus, the diversity of the trees can be enhanced by using all features. We tried different tree numbers and selected the one which gives the best predictive accuracy. The optimal tree number is 68. Table 3 shows that iPNHOT achieved higher values than the random forest model for all the six evaluation metrics, demonstrating that our two-step feature selection strategy and the SVM learning method are effective in predicting hot spot on protein-nucleic acid interfaces.

### Evaluation of our model on the independent test set

The generalization of the iPNHOT model was evaluated on the independent test set. Table 4 shows that the recall, the specificity, the accuracy on the independent test set is 0.571, 0.845, 0.815 that is close to the cross-validation recall, specificity, accuracy of 0.628, 0.913, 0.829, respectively, which shows the good generalization of the iPNHOT model.

**Table 4:** Comparison with mCSM-NA on both the training data set and the independent test set

Datasets	Methods	REC	PRE	SPE	ACC	F1 score	MCC
Training dataset	mCSM-NA	0.419	0.356	0.686	0.608	0.385	0.100
	iPNHOT	0.628	0.750	0.913	0.829	0.684	0.572
Training dataset (ProNIT)^a^	mCSM-NA	0.297	0.647	0.907	0.686	0.407	0.264
	iPNHOT	0.676	0.781	0.892	0.814	0.725	0.589
Independent test set	mCSM-NA	0.571	0.163	0.627	0.621	0.254	0.129
	iPNHOT	0.571	0.320	0.845	0.815	0.410	0.329

^a^The subset of the training data set which includes 102 residues collected from ProNIT

### Comparison with other methods

Our iPNHOT model is a single model which was built to predict the interface hot spot residues on both protein-RNA and protein-DNA interfaces. The SBHD server [11] is also for predicting hotspot residues on both protein-RNA and protein-DNA interfaces, however, it is not available now. mCSM-NA server [23] contains modules to predict mutagenic effect of residues on both protein-RNA or protein-DNA interfaces, and it is available to the community. HotSPRing [18] and PrabHot [24] are two models for predicting hot spots on protein-RNA interfaces. However, HotSPRing server does not work well because no results could be obtained for submitted jobs. PrabHot server only outputs the predicted scores for predicted hotspot residues. In addition, PrabHot defined the hotspot residues by using a cutoff value 1.0 kcal/mol, which is different from the cutoff value 2.0 kcal/mol used in this study. Thus, the AUROC and AUPRC are the only metrics that can be compared between PrabHot and iPNHOT. PrPDH [22] is a recently developed method for predicting hotspot on protein-DNA interfaces. In the method, the authors also defined the hot spot residues by using the cutoff value 1.0 kcal/mol. However, only 11 of the 32 residues in the independent test set that are on the protein-DNA interfaces are not used to train the PrPDH model, thus it is not suitable to compare our model with this method because of the small number of samples.

First, we compared our model to mCSM-NA on both the training data set and the independent test set. The prediction results for all examples in the training data set and the independent test set are shown in Table S2 and Table S4 (see Additional file 2), respectively. As shown in Table 4, the cross-validation results of iPNHOT outperform the predictive results of mCSM-NA according to all the 6 evaluation metrics. However, only part of the training data set, collected from ProNIT, has been used to train the mCSM-NA model. To fairly compare with mCSM-NA, we extracted the 102 interface residues obtained from ProNIT, and compared the predictive results between iPNHOT and mCSM-NA on these 102 data points. Table 4 indicates that iPNHOT outperforms mCSM-NA on all the metrics except specificity.

In addition, we also compared iPNHOT with mCSM-NA on the independent test set. Table 4 shows that iPNHOT outperforms mCSM-NA on all the metrics except recall.

**Fig. 7:**
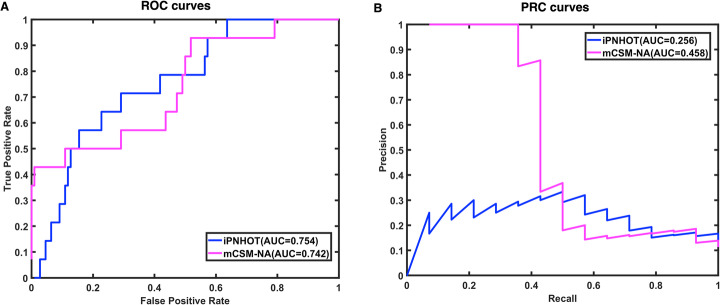
The ROC and PRC curves based on the predictive results of iPNHOT and mCSM-NA on the independent test set. **a**. ROC curves; **b**. PRC curves

In addition to the 6 performance metrics, we also plotted the ROC curves and PRC curves to compare different methods. Figure 5a shows the ROC curves based on the predictive results of mCSM-NA vs. iPNHOT on the training data set. For the 106 data collected from ProNIT, the area under the curve (AUROC) of mCSM-NA is 0.668 that is substantially lower than the AUROC of iPNHOT (0.831). Figure 7a shows that the AUROC of mCSM-NA is 0.742 which is lower than the AUROC of iPNHOT (0.754) on the independent test set. Figure 5b shows the PRC curves on the training data set. For the 106 data collected from ProNIT, the area under the PRC curve (AUPRC) of mCSM-NA is 0.590 that is substantially lower than the AUPRC of iPNHOT (0.730). Figure 7b shows that the AUPRC of mCSM-NA is 0.458 which is higher than the AUPRC of iPNHOT (0.256) on the independent test set.

According to the results mentioned above, our iPNHOT model is superior to mCSM-NA on 6 metrics including precision, specificity, accuracy, F1 score, MCC and AUROC, and mCSM-NA is superior to iPNHOT on only 1 metric that is AUPRC on the independent test set. As for PRC curve, although some researchers reported that PRC is suitable to evaluate the imbalanced dataset, others reported that the PRC curve are easily affected by the example with the largest output value [49], and the empirical PRC curve are highly imprecise estimate of the true curve, especially in the case of a small sample size and the class imbalance in favor of negative examples [50]. The PRC curve of our model on the independent test set demonstrates the opinions of the latter two papers. Thus, overall iPNHOT model outperforms the mCSM-NA model.

**Fig. 8:**
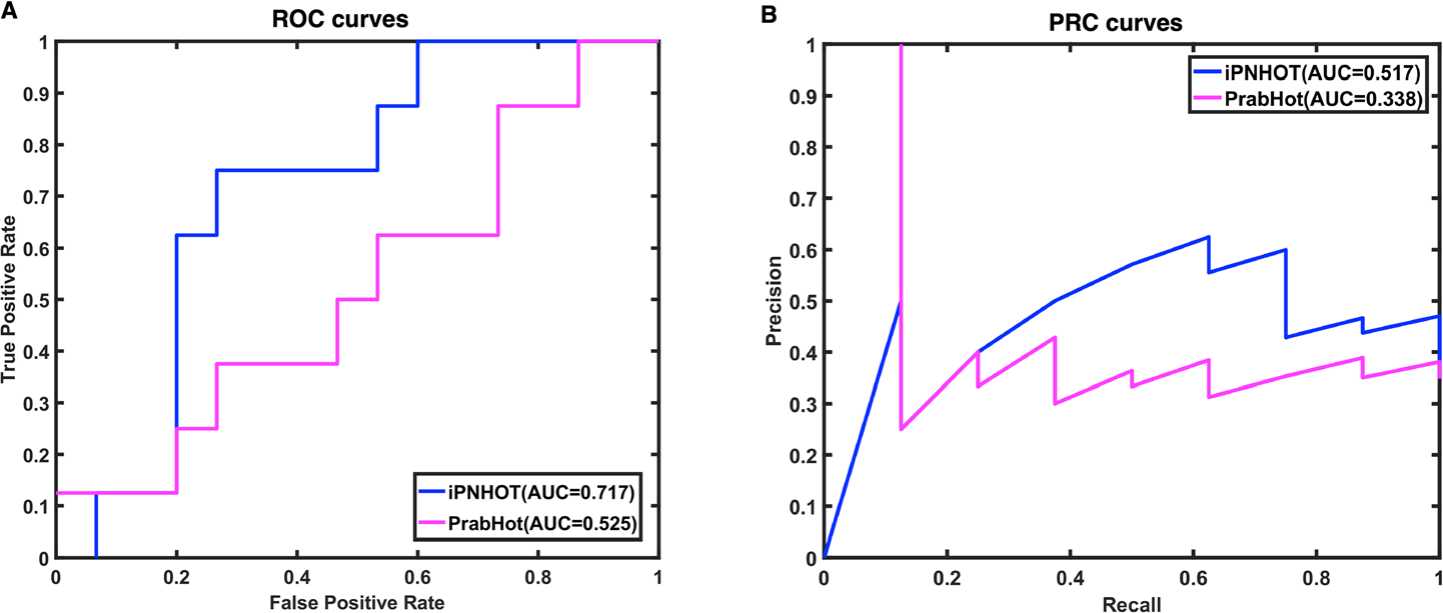
The ROC and PRC curves based on the predictive results of iPNHOT and PrabHot on the subset of the independent test set. **a**. ROC curves; **b**. PRC curves

In addition, we compared the AUROC and AUPRC between iPNHOT and the PrabHot. Because part of the data in the independent test set have been used to train the PrabHot model, the AUROC and AUPRC were calculated based on 23 samples which were not used to train the PrabHot model and whose predicted scores of PrabHot are available. As shown in Fig. 8a and b, the AUROC of PrabHot is 0.525 which is lower than the AUROC of iPNHOT (0.658) and the AUPRC of PrabHot is 0.338 which is also lower than the AUPRC of iPNHOT (0.517).

Thus, we demonstrated that our model outperforms other state-of-art methods for predicting hotspots on protein-nucleic acid interfaces.

**Fig. 9:**
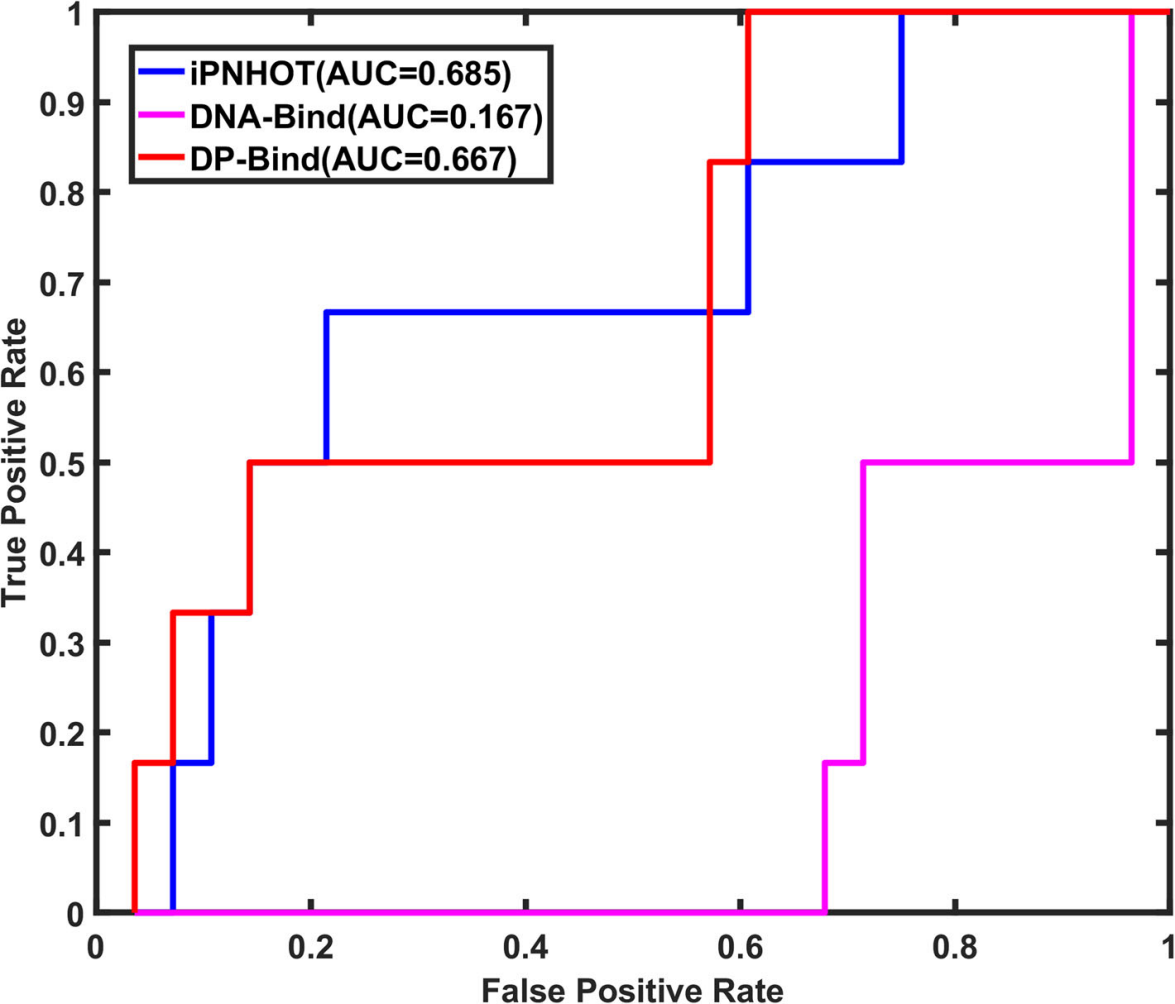
The ROC curves based on the predictive results of iPNHOT, DNA-Bind and DP-Bind on the protein-DNA interface samples in the independent test set

**Fig. 10:**
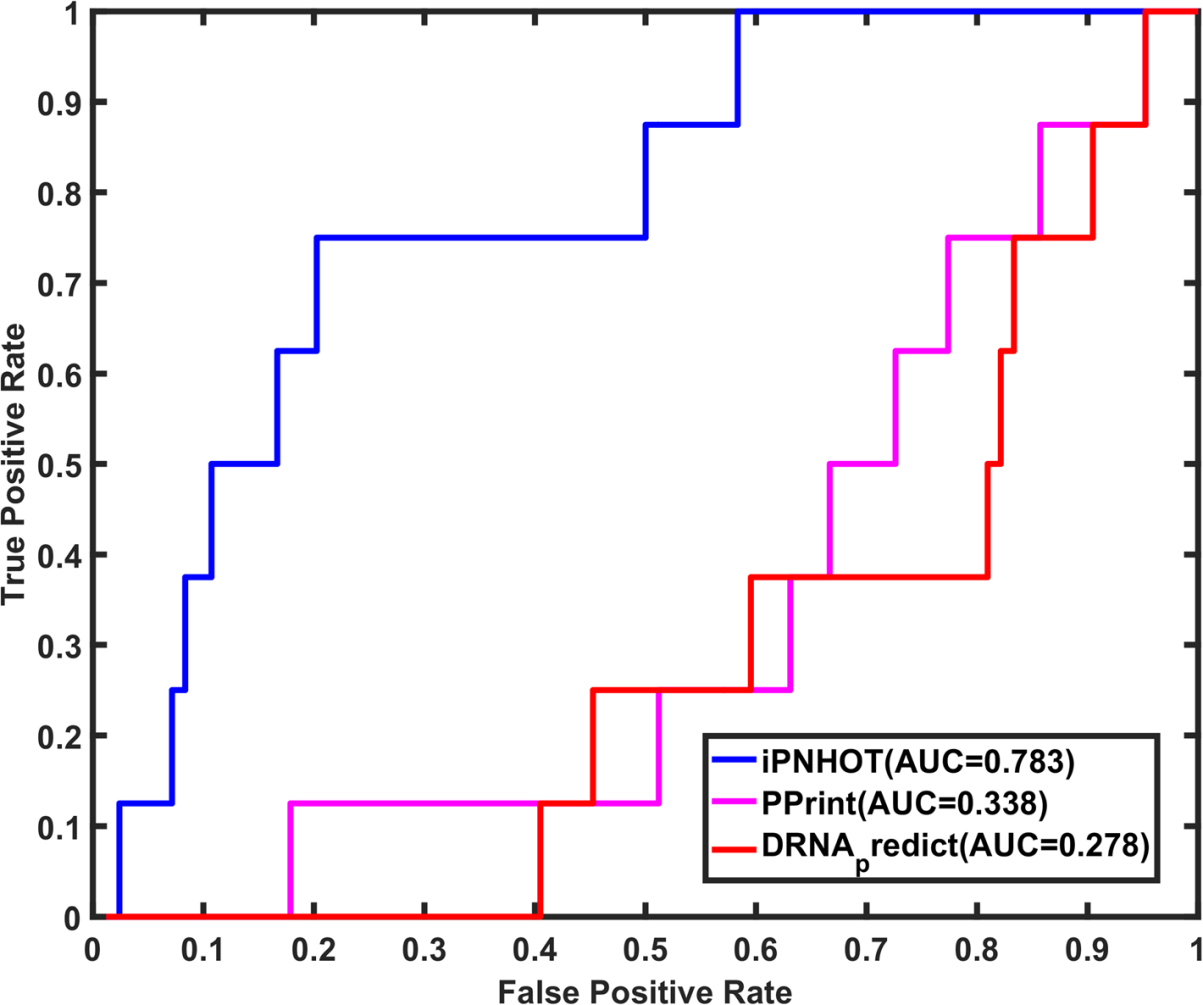
The ROC curves based on the predictive results of iPNHOT, Pprint and DRNApred on the protein-RNA interface samples in the independent test set

Furthermore, we also compared our method with two protein-DNA binding sites prediction methods and two protein-RNA binding sites prediction methods. As shown in Fig. 9, the AUROC of iPNHOT is 0.685 on protein-DNA interface samples of the independent test set, which is higher than the two protein-DNA binding sites prediction methods (0.167 for DNA-Bind [51] and 0.667 for DP-Bind [52]). Similarly, Fig. 10 shows that the AUROC of iPNHOT is 0.783 on protein-RNA interface samples of the independent test set, which is also higher than the two protein-RNA binding sites prediction methods (0.338 for Pprint [53] and 0.278 for DRNApred [54]).

### Post analysis of the selected features of the final model

To demonstrate the importance of the features used in the final model, we did a post analysis by removing one of the selected features and checking the performance of the models built based on the remaining features. As showed in Table 5, when we removed the feature Nphb, PItu, *∆DIs*, SAStau, *∆*SASsa^1/2^, *esp*3, and Helix respectively, the predictive accuracies decreased as expected. Especially, the predictive accuracies decreased substantially when *esp*3 was removed, which emphasizes the importance of this feature. The electrostatic complementarity on protein-DNA interfaces have been extensively reviewed in Harris et al.’s article [55]. Although it is still a controversy for the contribution of electrostatic potential to the binding affinity, our results indicate that the electrostatic potential can be a useful feature for predicting hotspots on protein-RNA/DNA interfaces.

**Table 5:** Predictive results of the models built by removing one of the selected features

Feature removed	REC	PRE	SPE	ACC	F1 score	MCC
Nphb	0.535	0.605	0.855	0.761	0.568	0.405
PItu	0.570	0.700	0.899	0.802	0.628	0.5
*∆DIs*	0.442	0.655	0.903	0.768	0.528	0.395
SAStau	0.593	0.761	0.923	0.826	0.667	0.559
*∆*SASsa^1/2^	0.570	0.690	0.894	0.799	0.624	0.493
*esp*3	0.430	0.597	0.879	0.747	0.500	0.345
Helix	0.523	0.634	0.874	0.771	0.573	0.423

### Statistical analysis of the selected 7 features

**Fig. 11:**
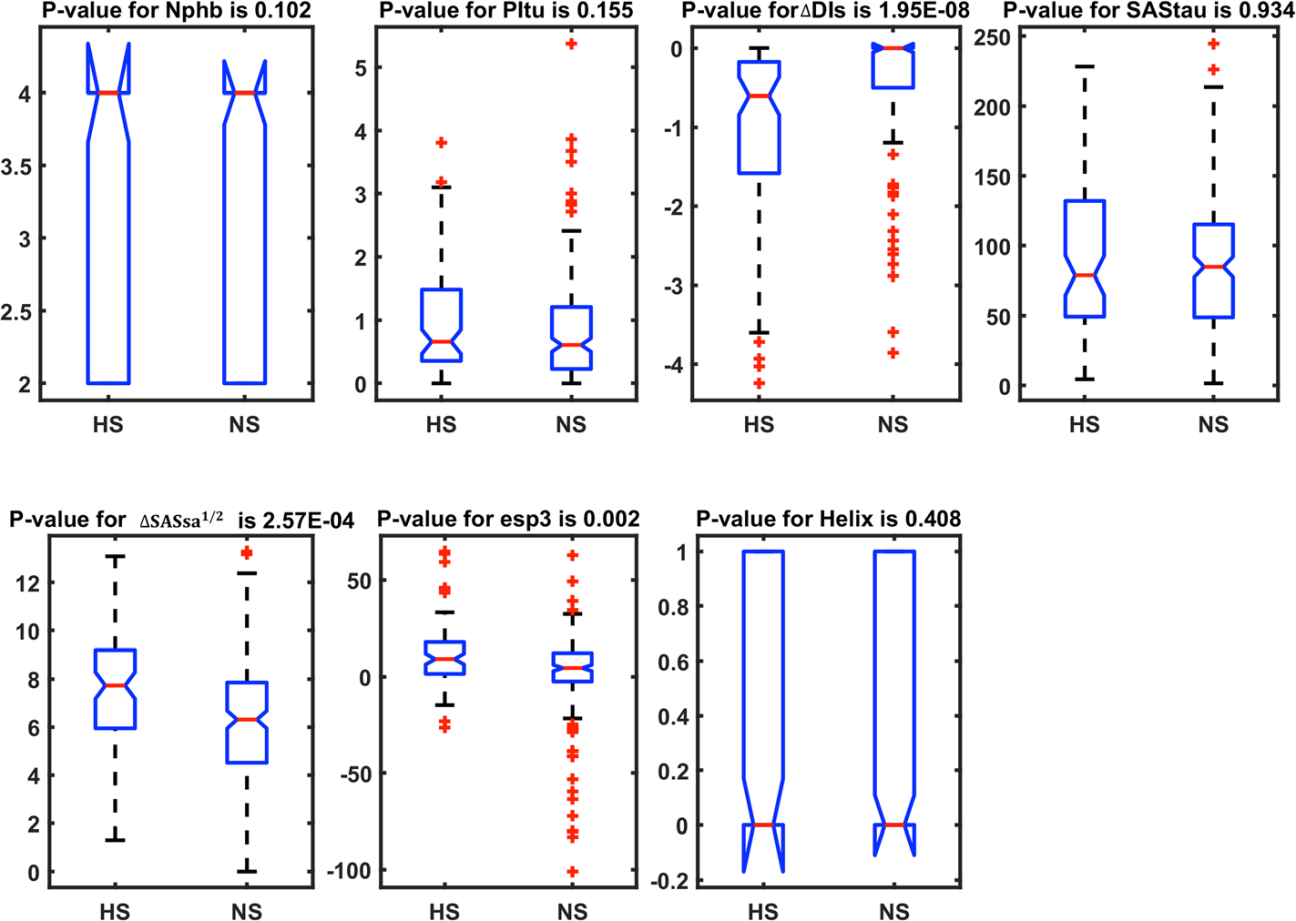
Box-plots and the *P*-values of the 9 selected features

To further evaluate the ability of the 7 selected features to distinguish hot spot from non-hot spots, we used the Wilcoxon rank sum analysis. Figure 11 shows that three of the 7 selected features can significantly differentiate hot spots from non-hot spots with *p*-values less than 0.05, which are *∆DIs*, *∆*SASsa^1/2^, and esp3. The first features, *∆DI*, reflect the shape complementarity between protein residues and nucleic acid upon binding. As we proposed in the “Feature extraction” section, *∆*SASsa^1/2^ may related to the desolvation energy upon binding. As for esp3, it is the electrostatic potential of protein surface patch around the target residue. For hot spots, the average value of the feature is 11.6 compared to 1.77 for non-hot spots. Because of the negative electrostatic potential of nucleic acid surface, this feature may partially reflect the electrostatic potential complementarity between the protein surface patch and the nucleic acid surface patch around the target residue. Thus, these 3 features combined the effects of shape complementarity, electrostatic potential complementarity, and the desolvation energy.

In addition to the features that were statistically important on an individual basis, the other 4 of the 7 selected features were also kept in the final model. This suggests the possibility of coordinated effects between different features. In particular, a feature that is not individually significant can gain significance when combined with other information gleaned from other features.

Moreover, the analysis (Figure S1-S6) of the 20 selected features by decision tree can be found in the Additional file 1.

### Case study

**Fig. 12:**
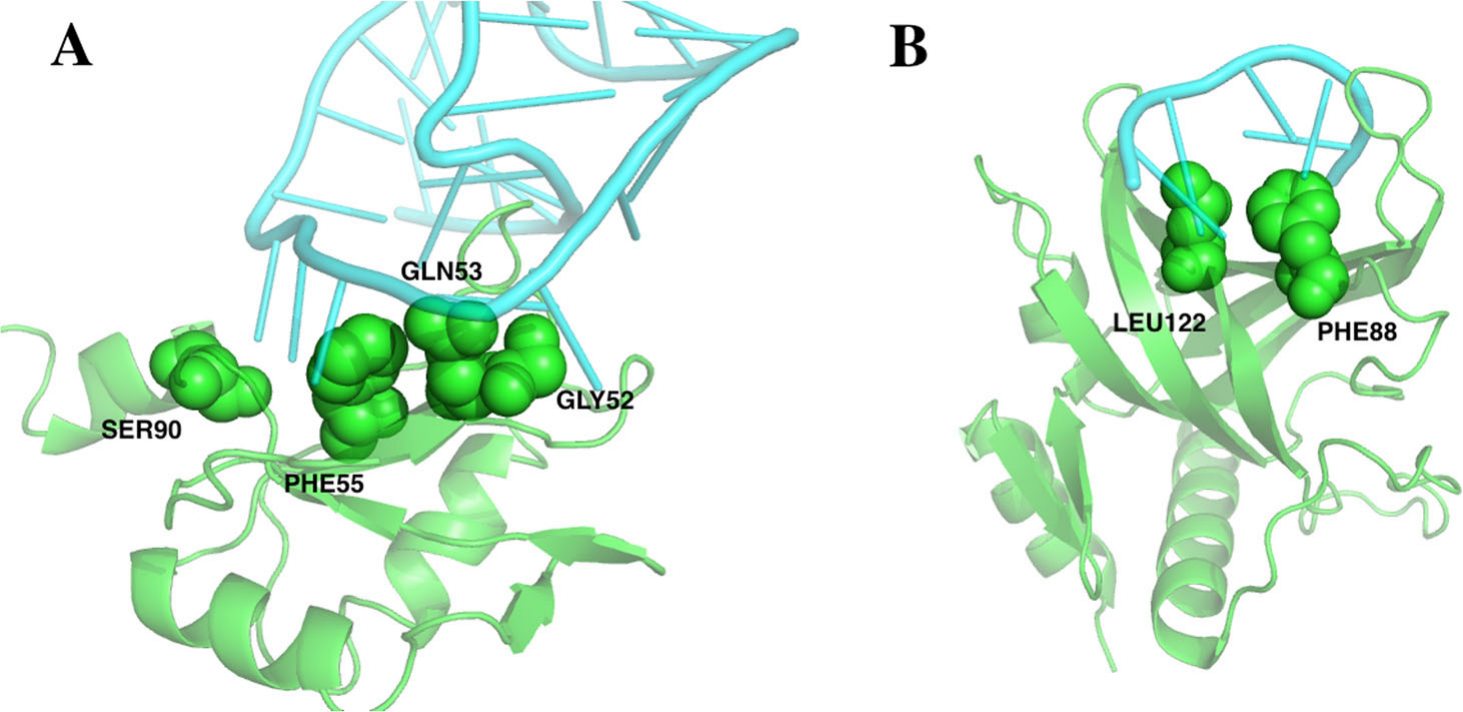
Interface hotspot residues of U1 small nuclear ribonucleoprotein A (U1A) and an RNA and Pot1(protection of telomere) and a DNA. **a**. 1AUD; **b**. 1QZG

To visualize the hotspot residues on the protein-nucleic acid interfaces, we plotted two cases by using PyMol. The first one is the complex of U1 small nuclear ribonucleoprotein A (U1A) and an RNA, for which the PDB ID is 1AUD. As shown in Fig. 12a, 4 hotspot residues and 2 non-hotspot residues at the interface had been recorded in the training dataset. Our model identified all the 4 hot spot residues as hotspots and the 2 non-hotspot residues as non-hotspot residues when doing both leave one out cross validation and leave one-protein out cross validation. On the contrary, mCSM-NA did not assign any of the 4 residue as hot spot residue. The second case is the complex of Pot1(protection of telomere) and a DNA, for which the PDB ID is 1QZG. As shown in Fig. 12b, 2 hotspot residues and 3 non-hotspot residues at the interface had been recorded in the training data set. Our model identified all of the 2 hotspot residues as hotspots and all the 3 non-hotspot residues as non-hotspot residues when doing both leave one out cross validation and leave one-protein out cross validation. However, mCSM-NA did not detect any of the 2 hot spot residues. Note that both 1AUD and 1QZG were collected from ProNIT, which had been used to train the mCSM-NA model.

## Conclusion

The interface hot spot residues provide clues to understand the principles driving the interaction between protein and nucleic acids. In this study, we collected a non-redundant training dataset with 293 alanine-mutated residues on protein-nucleic acid interfaces from dbAMEPNI database. Based on this data set, we developed a single knowledge-based method to predict hot spot residues on both protein-DNA and protein-RNA interfaces. Using the two-step feature selection strategy, we selected 7 features from the original 97 features, which include some unique feature such as *∆*SASsa^1/2^, and esp3. Our model shows better performances compared with mCSM-NA on both the training data set and the independent test set.

The selected features were further analyzed to reveal the relationship between features and hot spots. Among the selected 7 features, the differences of 3 features for hot spot and non-hot spot residues are statistically significant and the 3 features are *∆DIs*, *∆*SASsa^1/2^, and esp3. The features, *∆DIs*, reflect the shape complementarity or the buried condition of the target residues. The *∆*SASsa^1/2^ may reflect the desolvation energy of residues. The esp3 reflect the patch electrostatic potential complementarity around the residue. The differences of the other 4 features are not significant and the 4 features are Nphb, SAStau, and Helix. Our results show both predictive ability of single feature and the complementarity between features are important for building our model.

## Supplementary information

**Additional file 1. **Supplementary Materials for iPNHOT: A knowledge-based approach for identifying protein-nucleic acid interaction hot spots. This file provides more detailed data for protein-nucleic acids complexes, all the features generated in this study, and other tables for analysis and discussion. **Table S1**: Protein-nucleic acid complexes in the training dataset. **Table S3**: Protein-nucleic acid complexes in the independent test set. **Table S5**: All features generated for building our model to predict hotspot on protein-NA interfaces. **Table S6**: The numerical values of 10 different kinds of properties of the 20 amino acids. **Table S7:** Features selected and the corresponding cross validation performance in the SFS process. **Table S8**: The features selected and the corresponding cross validation performance in the SFS process based on the original 97 features. **Description of Statistically analysis of the correlations between hotspots and different features:** This section also includes 6 figures (Figure S1-S6) which visually show the results of the statistically analysis of the 20 features selected by decision tree.

**Additional file 2 **. Datasets for iPNHOT: A knowledge-based approach for identifying protein-nucleic acid interaction hot spots. This file provides more detailed data for the datasets. **Table S2**: The interface residues with observed ∆∆퐺 values of the training data set. **Table S4:** The interface residues with observed ∆∆퐺 values of the medium test set.

## Data Availability

The webserver is at http://zhulab.ahu.edu.cn/iPNHOT/. The two data sets used in this study are included in the Additional file 2. All the other data generated or analyzed during this study are included in this published article or the Additional files.

## Abbreviations

SVM: Support Vector Machine
HS: Hotspots
GA: Genetic Algorithm
DI: Depth Index
PI: Protrusion Index
SASA: Solvent Accessible Surface Area
PSSM: Position Specific Scoring Matrix
SEN: Sensitivity
SPE: Specificity
ACC: Accuracy
PRE: Precision
MCC: Matthew Correlation Coefficient
AUC: Area Under the Curve
ROC: Receiver Operating Characteristic
SFS: Sequential Forward feature Selection
