# Supplementary material for: iPNHOT: a knowledge-based approach for identifying protein-nucleic acid interaction hot spots
Source: BMC Bioinformatics. 2020 Jul 6;21:289. doi: 10.1186/s12859-020-03636-w (PMC7336410; doi:10.1186/s12859-020-03636-w)
Supplement: Supplementary file 1 — Additional file 1. Supplementary Materials for iPNHOT: A knowledge-based approach for identifying protein-nucleic acid interaction hot spots. This file provides more detailed data for protein-nucleic acids complexes, all the features generated in this study, and other tables for analysis and discussion. Table S1: Protein-nucleic acid complexes in the training dataset. Table S3: Protein-nucleic acid complexes in the independent test set. Table S5: All features generated for building our model to predict hotspot on protein-NA interfaces. Table S6: The numerical values of 10 different kinds of properties of the 20 amino acids. Table S7: Features selected and the corresponding cross validation performance in the SFS process. Table S8: The features selected and the corresponding cross validation performance in the SFS process based on the original 97 features. Description of Statistically analysis of the correlations between hotspots and different features: This section also includes 6 figures (Figure S1-S6) which visually show the results of the statistically analysis of the 20 features selected by decision tree. [file 12859_2020_3636_MOESM1_ESM.pdf]

Supplementary Materials for

iPNHOT: A knowledge-based approach for identifying protein-nucleic acid interaction hot spots

Xiaolei Zhu[1,2]* Ling Liu[2], Jingjing He[2], Ting Fang[2], Yi Xiong[3] and Julie C. Mitchell[4]*
[1] School of Sciences, Anhui Agricultural University, Hefei, Anhui 230036, China
[2] School of Life Sciences, Anhui University, Hefei, Anhui 230601, China
[3] School of Life Sciences and Biotechnology, Shanghai Jiao Tong University, Shanghai 200240, China
[4] Biosciences Division, Oak Ridge National Laboratory, Oak Ridge TN 37830
Contact: xlzhu_mdl@hotmail.com; mitchelljc@ornl.gov

Table S1. Protein-nucleic acid complexes in the training dataset.

PDBID	Protein Names	Types of Nucleic Acids
1AAY	ZIF268 ZINC FINGER	dsDNA
1AIS	TATA-BINDING PROTEIN	dsDNA
1AUD	U1 SMALL NUCLEAR RIBONUCLEOPROTEIN A (U1A)	ssRNA
1AZ0	ENDONUCLEASE ECORV	dsDNA
1B3T	EBNA-1 NUCLEAR PROTEIN	dsDNA
1BHM	RESTRICTION ENDONUCLEASE BAMHI	dsDNA
1C9S	TRP RNA-BINDING ATTENUATION PROTEIN	ssRNA
1ECR	ESCHERICHIA COLI REPLICATION TERMINATOR PROTEIN (TUS)	dsDNA
1EWQ	DNA MISMATCH REPAIR PROTEIN MUTS	dsDNA
1J5N	NONHISTONE CHROMOSOMAL PROTEIN 6A	dsDNA
1JMC	HUMAN REPLICATION PROTEIN A	ssDNA
1MHT	HHAI METHYLTRANSFERASE	dsDNA
1MSE	C-MYB DNA-BINDING DOMAIN	dsDNA
1PNR	PURINE REPRESSOR	ssDNA
1QFQ	36-MER N-TERMINAL PEPTIDE OF THE N PROTEIN	ssRNA
1QRV	HIGH MOBILITY GROUP PROTEIN D	dsDNA
1QZG	PROTECTION OF TELOMERES PROTEIN 1	ssDNA
1RUN	CATABOLITE GENE ACTIVATOR PROTEIN (CAP)	dsDNA
1TN9	TN916 INTEGRASE N-TERMINAL DOMAIN	dsDNA
1VS5	30S RIBOSOMAL PROTEIN	dsDNA
2A0I	DNA HELICASE I	ssDNA
2BPF	RAT DNA POLYMERASE BETA	dsDNA
2ERR	ATAXIN-2-BINDING PROTEIN 1	ssRNA
2G1P	DNA ADENINE METHYLTRANSFERASE	dsDNA
2I05	ESCHERICHIA COLI REPLICATION TERMINATOR PROTEIN	dsDNA
2VS7	HOMING ENDONUCLEASE I-DMOI	dsDNA

2VYE	REPLICATIVE DNA HELICASE	ssDNA
2Y8W	CRISPR ENDORIBONUCLEASE CSE3	ssRNA
3GPX	DNA GLYCOSYLASE	dsDNA
3K5Y	FEM-3 MRNA-BINDING FACTOR 2	ssRNA
3NCI	DNA POLYMERAS	dsDNA
3NH0	RIBONUCLEASE T	ssDNA
3OD8	HUMAN PARP-1 ZINC FINGER 1	dsDNA
3ODC	HUMAN PARP-1 ZINC FINGER 2	dsDNA
3OSG	MYB-LIKE DNA-BINDING DOMAIN CONTAINING PROTEIN	dsDNA
3PVV	CHROMOSOMAL REPLICATION INITIATOR PROTEIN DNAA	dsDNA
3QMG	CPG-BINDING PROTEIN	dsDNA
3QSU	RNA CHAPERONE HFQ	ssRNA
3RN2	INTERFERON-INDUCIBLE PROTEIN AIM2	dsDNA
3RW6	NUCLEAR RNA EXPORT FACTOR 1	dsRNA
3SPD	APRATAXIN-LIKE PROTEIN	dsDNA
3SZQ	APRATAXIN-LIKE PROTEIN	ssDNA
3U4M	50S RIBOSOMAL PROTEIN L1	ssRNA
3WPC	TOLL-LIKE RECEPTOR 9	ssDNA
3WTS	RUNT-RELATED TRANSCRIPTION FACTOR 1	dsDNA
4ALP	LIN28 ISOFORM B	ssRNA
4B5F	PUTATIVE EXODEOXYRIBONUCLEASE	dsDNA
4BNC	HUMAN ETV1	dsDNA
4DQI	DNA POLYMERASE I	dsDNA
4ED5	ELAV-LIKE PROTEIN 1	ssRNA
4GZN	ZINC FINGER PROTEIN 57	dsDNA
4HF1	HTH-TYPE TRANSCRIPTIONAL REGULATOR ISCR	dsDNA
4HN5	HTH-TYPE TRANSCRIPTIONAL REGULATOR ISCR	dsDNA
4HQB	SINGLE-STRANDED DNA-BINDING PROTEIN DDRB	ssDNA
4HT8	PROTEIN HFQ	ssRNA
4L5R	INTERFERON-ACTIVABLE PROTEIN 202	dsDNA
4M9E	KRUEPPEL-LIKE FACTOR 4	dsDNA
4NGD	ENDORIBONUCLEASE DICER	dsDNA
4NKU	POLY(A) RNA POLYMERASE PROTEIN CID1	ssRNA
4OOG	RIBONUCLEASE 3	dsRNA
4QJU	DNA-BINDING PROTEIN HU	dsDNA
4QTJ	WHITE-OPAQUE REGULATOR 1	dsDNA
4QVC	RNA-BINDING PROTEIN HFQ	ssRNA
4R3I	YTH DOMAIN-CONTAINING PROTEIN 1	ssRNA
4R56	CHROMATIN PROTEIN CREN7	dsDNA
4R8I	C-C MOTIF CHEMOKINE 2	dsRNA
4RCJ	YTH DOMAIN-CONTAINING FAMILY PROTEIN 1	ssRNA
4RKG	E3 UBIQUITIN-PROTEIN LIGASE MSL-2	dsDNA
4TMU	RECQ	ssDNA
4WB2	COMPLEMENT C5	DNA/RNA

4WCG	ORF112	ssDNA
4X5V	DNA POLYMERASE LAMBDA	dsDNA
4XQ8	DNA POLYMERASE LAMBDA	dsDNA
4XR0	DNA REPLICATION TERMINUS SITE-BINDING PROTEIN	dsDNA
4ZSF	BSAWI ENDONUCLEASE	ssDNA
5AWH	UNCHARACTERIZED PROTEIN	dsDNA
5CO8	NUCLEASE-LIKE PROTEIN	dsDNA
5D8C	MERR FAMILY REGULATOR PROTEIN	dsDNA
5DET	RNA-BINDING PROTEIN WITH MULTIPLE SPLICING	ssRNA
5DFF	DNA-(APURINIC OR APYRIMIDINIC SITE) LYASE	dsDNA
5DNO	YTH DOMAIN-CONTAINING PROTEIN MMI1	ssRNA
5DWB	TYPE-2 RESTRICTION ENZYME AGEI	dsDNA
5ED4	RESPONSE REGULATOR	dsDNA
5EIM	YTH DOMAIN-CONTAINING PROTEIN MMI1	ssRNA
5ELK	RING FINGER PROTEIN UNKEMPT HOMOLOG	ssRNA
5EXH	METHYLCYTOSINE DIOXYGENASE TET3	dsDNA
5F55	SINGLE-STRANDED-DNA-SPECIFIC EXONUCLEASE	ssDNA
5FD3	PROTEIN LIN-54 HOMOLOG	dsDNA
5GXH	GEM-ASSOCIATED PROTEIN 5	ssRNA
5H1K	GEM-ASSOCIATED PROTEIN 5	ssRNA
5III	DNA POLYMERASE LAMBDA	ds/ssDNA
5JBJ	LGP2	dsRNA
5KUB	DNA-7-METHYLGUANINE GLYCOSYLASE	dsDNA
5M0J	SWI5-DEPENDENT HO EXPRESSION PROTEIN 2/3	ssRNA
5M3H	POLYMERASE ACIDIC PROTEIN	dsRNA
5SZX	ZTA TRANSCRIPTION FACTOR	dsDNA
5T5C	NUCLEASE EXOG	dsDNA
5TWP	DNA-DIRECTED DNA/RNA POLYMERASE MU	dsDNA
5U2R	DNA POLYMERASE BETA	dsDNA
5U8G	DNA POLYMERASE BETA	dsDNA
5UDZ	PROTEIN LIN-28 HOMOLOG A	dsRNA
5W1H	LBACAS13A	ssRNA
5WVW	CHROMATIN PROTEIN CREN7	dsDNA
5WWX	RNA-BINDING E3 UBIQUITIN-PROTEIN LIGASE MEX3C	ssRNA
5XFQ	PHD FINGER PROTEIN 1	dsDNA

Table S3. Protein-nucleic acid complexes in the independent test set.

PDBID	Protein Names	Type of Nucleic Acids
1ASY	ASPARTYL-TRNA SYNTHETASE	dsRNA
1JBS	RIBOTOXIN RESTRICTOCIN	dsRNA
1U0B	CYSTEINYL-TRNA SYNTHETASE	ssRNA
1YVP	RO RIBONUCLEOPROTEIN	dsRNA
2BX2	RIBONUCLEASE E	ssRNA
2IX1	EXORIBONUCLEASE 2	ssRNA
2PJP	SELENOCYSTEINE-SPECIFIC ELONGATION FACTOR	ssRNA
2ZI0	PROTEIN 2B	ssRNA
2ZKO	NON-STRUCTURAL PROTEIN 1	dsRNA
2ZZM	UNCHARACTERIZED PROTEIN MJ0883	ssRNA
3EQT	ATP-DEPENDENT RNA HELICASE DHX58	dsRNA
3MOJ	ATP-DEPENDENT RNA HELICASE DBPA	ssRNA
1FEU	50S RIBOSOMAL PROTEIN L25	dsRNA
1ZDI	RNA BACTERIOPHAGE MS2 COAT PROTEIN	ssRNA
2KXN	TRANSFORMER-2 PROTEIN HOMOLOG BETA	ssRNA
2XB2	REGULATOR OF NONSENSE TRANSCRIPTS 3B	ssRNA
3AM1	L-SERYL-TRNA(SEC) KINASE	dsRNA
3UZS	BETA-ADRENERGIC RECEPTOR KINASE 1	dsRNA
3VYY	ATP-DEPENDENT RNA HELICASE A	dsRNA
4CIO	PROTEIN SUP-12	ssRNA
4G0A	NON-STRUCTURAL PROTEIN 2	ssRNA
5EV1	SPLICING FACTOR U2AF	DNA/RNA
5HO4	HETEROGENEOUS NUCLEAR RIBONUCLEOPROTEINS A2/B1	ssRNA
5VMV	TRANSCRIPTIONAL REGULATOR KAISO	dsDNA
5ZLN	TOLL-LIKE RECEPTOR 9	ssDNA
6C1A	METHYL-CPG-BINDING DOMAIN PROTEIN 2	dsDNA
6EN0	INT PROTEIN	dsDNA
1BPX	DNA POLYMERASE BETA	dsDNA
1FOS	TWO HUMAN C-FOS:C-JUN	dsDNA
1HCQ	THE ESTROGEN RECEPTOR DNA-BINDING DOMAIN	dsDNA
2MXF	The C-Terminal domain of MvaT	dsDNA
3UFD	Restriction-modification controller proteins	dsDNA

Table S5. All features generated for building our model to predict hotspot on protein-NA interfaces.

No.	Feature description	Symbol
1	Number of atoms	Na
2	Number of electrostatic charge	Nec
3	Number of potential hydrogen bonds	Nphb
4	Hydrophobicity	Hdrpo
5	Hydrophilicity	Hdrpi
6	Propensity	Prop
7	Isoelectric point	Isoep
8	Mass	Mass
9	Expected number of contacts within 14Å sphere	Enc
10	Electron-ion interaction potential	Eiip
11	Average depth index of the total residue in unbound state	DItu
12	Average depth index of the side chain of the residue in unbound state	DIsu
13	Average protrusion index of the total residue in unbound state	PItu
14	Average protrusion index of the side chain of the residue in unbound state	PIsu
15	Average depth index of the total residue in bound state	DItb
16	Average depth index of the side chain of the residue in bound state	DIsb
17	Average protrusion index of the total residue in bound state	PItb
18	Average protrusion index of the side chain of the residue in bound state	PIsb
19	$DItu - DItb$	ΔDIt
20	$DIsu - DIsb$	ΔDIs
21	$PItu - PItb$	ΔPIt
22	$PIsu - PIsb$	ΔPIs
23	$(DItu - DItb)/DItu$	relDIt
24	$(DIsu - DIsb)/DIsu$	relDIs
25	$(PItu - PItb)/PItu$	relPIt
26	$(PIsu - PIsb)/PIsu$	relPIs
27	$(SAStau - SAStab)/SAStau$	relSAS
28	$(SAStru - SAStrb)/SAStau$	relSASrel
29	Total absolute solvent accessible surface area in unbound state	SAStau
30	Total relative solvent accessible surface area in unbound state	SAStru
31	Side chain absolute solvent accessible surface area in unbound state	SASsau
32	Side chain relative solvent accessible surface area in unbound state	SASsru
33	Backbone absolute solvent accessible surface area in unbound state	SASbau
34	Backbone relative solvent accessible surface area in unbound state	SASbru
35	Non polar absolute solvent accessible surface area in unbound state	SASnau
36	Non polar relative solvent accessible surface area in unbound state	SASnru
37	Polar absolute solvent accessible surface area in unbound state	SASpau
38	Polar relative solvent accessible surface area in unbound state	SASpru
39	Total absolute solvent accessible surface area in bound state	SAStab
40	Total relative solvent accessible surface area in bound state	SAStrb
41	Side chain absolute solvent accessible surface area in bound state	SASsab
42	Side chain relative solvent accessible surface area in bound state	SASsrb
43	Backbone absolute solvent accessible surface area in bound state	SASbab
44	Backbone relative solvent accessible surface area in bound state	SASbrb
45	Non polar absolute solvent accessible surface area in bound state	SASnab
46	Non polar relative solvent accessible surface area in bound state	SASnrb
47	Polar absolute solvent accessible surface area in bound state	SASpab
48	Polar relative solvent accessible surface area in bound state	SASprb
49	$(\mathrm{SAStau} - \mathrm{SAStab})^{1/2}$	$\Delta\mathrm{SASta}^{1/2}$
50	$(\mathrm{SASsau} - \mathrm{SASsab})^{1/2}$	$\Delta\mathrm{SASsa}^{1/2}$
51	$(\mathrm{SASpau} - \mathrm{SASpab})^{1/2}$	$\Delta\mathrm{SASpa}^{1/2}$
52	$(\mathrm{SASnau} - \mathrm{SASnab})^{1/2}$	$\Delta\mathrm{SASna}^{1/2}$
53	$SAStau - SAStab$	ΔSASta
54	$SASsau - SASsab$	ΔSASsa
55	$SASpau - SASpab$	ΔSASpa
56	$SASnau - SASnab$	ΔSASna
57	$(\mathrm{SAStau} - \mathrm{SAStab})^{3/2}$	$\Delta\mathrm{SASta}^{3/2}$
58	$(\mathrm{SASsau} - \mathrm{SASsab})^{3/2}$	$\Delta\mathrm{SASsa}^{3/2}$
59	$(\mathrm{SASpau} - \mathrm{SASpab})^{3/2}$	$\Delta\mathrm{SASpa}^{3/2}$
60	$(\mathrm{SASnau} - \mathrm{SASnab})^{3/2}$	$\Delta\mathrm{SASna}^{3/2}$
61	$(\mathrm{SAStau} - \mathrm{SAStab})^{2}$	$\Delta\mathrm{SASta}^{2}$

62	$(\mathrm{SASsau} - \mathrm{SASsab})^2$	$\Delta\mathrm{SASsa}^2$
63	$(\mathrm{SASpau} - \mathrm{SASpab})^2$	$\Delta\mathrm{SASpa}^2$
64	$(\mathrm{SASnau} - \mathrm{SASnab})^2$	$\Delta\mathrm{SASna}^2$
65	$(\mathrm{SAStru} - \mathrm{SAStrb})^{1/2}$	$\Delta\mathrm{SAStr}^{1/2}$
66	$(\mathrm{SASsru} - \mathrm{SASsrb})^{1/2}$	$\Delta\mathrm{SASsr}^{1/2}$
67	$(\mathrm{SASpru} - \mathrm{SASprb})^{1/2}$	$\Delta\mathrm{SASpr}^{1/2}$
68	$(\mathrm{SASnru} - \mathrm{SASnrb})^{1/2}$	$\Delta\mathrm{SASnr}^{1/2}$
69	$SAStru - SAStrb$	$\Delta\mathrm{SAStr}$
70	$SASsru - SASsrb$	$\Delta\mathrm{SASsr}$
71	$SASpru - SASprb$	$\Delta\mathrm{SASpr}$
72	$SASnru - SASnrb$	$\Delta\mathrm{SASnr}$
73	$(\mathrm{SAStru} - \mathrm{SAStrb})^{3/2}$	$\Delta\mathrm{SAStr}^{3/2}$
74	$(\mathrm{SASsru} - \mathrm{SASsrb})^{3/2}$	$\Delta\mathrm{SASsr}^{3/2}$
75	$(\mathrm{SASpru} - \mathrm{SASprb})^{3/2}$	$\Delta\mathrm{SASpr}^{3/2}$
76	$(\mathrm{SASnru} - \mathrm{SASnrb})^{3/2}$	$\Delta\mathrm{SASnr}^{3/2}$
77	$(\mathrm{SAStru} - \mathrm{SAStrb})^2$	$\Delta\mathrm{SAStr}^2$
78	$(\mathrm{SASsru} - \mathrm{SASsrb})^2$	$\Delta\mathrm{SASsr}^2$
79	$(\mathrm{SASpru} - \mathrm{SASprb})^2$	$\Delta\mathrm{SASpr}^2$
80	$(\mathrm{SASnru} - \mathrm{SASnrb})^2$	$\Delta\mathrm{SASnr}^2$
81	Electrostatic potential of the residue	$esp1$
82	Electrostatic potential of the neighbor residue of the target residue	$esp2$
83	Electrostatic potential of the neighbor residue and the target residue	$esp3$
84	Average of esp2	$esp4$
85	Average of esp3	$esp5$
86	The number of hydrogen bond formed by the residue and the nucleic acids	HB1
87	The number of hydrogen bond between side chain of the residue and the nucleic acids	HB2
88	If the secondary structure of the residue is alpha helix (H) or not	Helix
89	If the secondary structure of the residue is beta-sheet (E) or not	Sheet
90	If the secondary structure of the residue is a turn (B,T,S) or not	Turn
91	If the secondary structure of the residue is other helix (G,I) or not	Helix1
92	If the secondary structure of the residue is loops or not	Loop
93	Conservation score	CNSV
94	Relative conservation of the actual residue compared to the alanine on a certain position based on the weighted observed percentages	CNSV_REL1_wop
95	Relative conservation of the residue with maximum percentage compared to the alanine on a certain position based on the weighted observed percentages	CNSV_REL2_wop
96	Relative conservation of the actual residue compared to the alanine on a certain position based on the position-specific scores	CNSV_REL1_pss
97	Relative conservation of the residue with maximum percentage compared to the alanine on a certain position based on the position-specific scores	CNSV_REL2_pss

Table S6. The numerical values of 10 different kinds of properties of the 20 amino acids

Residue	Na [a]	Nec	Nphb	Hdrpo	Hdrpi	Prop	Isoep	Mass	Enc	Eiip
A	5	0	2	0.25	3	-0.17	6.11	71.1	-22	0.0373
C	6	0	2	0.04	-1	0.43	6.31	103.1	4.66	0.0829
D	8	-1	4	-0.72	3	-0.38	5.945	115.1	-4.12	0.1263
E	9	-1	4	-0.62	3	-0.13	5.785	129.1	-3.64	0.0058
F	11	0	2	0.61	-2.5	0.82	5.755	147.2	5.27	0.0946
G	4	0	2	0.16	0	-0.07	6.065	57	-1.62	0.005
H	10	0	4	-0.4	-0.5	0.41	5.565	137.1	1.28	0.0242
I	8	0	2	0.73	-1.8	0.44	6.04	113.2	5.58	0
K	9	1	2	-1.1	3	-0.36	5.61	128.2	-4.18	0.0371
L	8	0	2	0.53	-1.8	0.4	6.035	113.2	5.01	0
M	8	0	2	0.26	-1.3	0.66	5.705	131.2	3.51	0.0823
N	8	0	4	-0.64	0.2	0.12	5.43	114.1	-2.65	0.0036
P	7	0	2	-0.07	0	-0.25	6.295	97.1	-3.03	0.0198
Q	9	0	4	-0.69	0.2	-0.11	5.65	128.1	-2.76	0.0761
R	11	1	4	-1.76	-0.5	0.27	5.405	156.2	-0.93	0.0959
S	6	0	4	-0.26	0.3	-0.33	5.7	87.1	-2.84	0.0829
T	7	0	4	-0.18	-0.4	-0.18	5.595	101.1	-1.2	0.0941
V	7	0	2	0.54	-1.5	0.27	6.015	99.1	4.45	0.0057
W	14	0	3	0.37	-3.4	0.83	5.935	186.2	52	0.0548
Y	12	0	3	0.02	-2.3	0.66	5.705	163.2	2.15	0.0516

[a] The explanation of the 10 properties can be found in Table S5.

Table S7. Features selected and the corresponding cross validation performance in the SFS process.

Round	Features selected[a]	Recall	Precision	F1 score
	ΔDIs (20)	0.291	0.641	0.400
	$\Delta SASsa^{1/2}$ (50)	0.361	0.304	0.330
	ΔDIt (19)	0.256	0.349	0.295
2	ΔDIs (20), $\Delta SASsa^{1/2}$ (50)	0.454	0.609	0.520
	ΔDIs (20), $\Delta SASta^{1/2}$ (49)	0.419	0.655	0.511
	Nphb (3), ΔDIs (20)	0.361	0.674	0.470
3	Hydrophobicity (4), ΔDIs (20), $\Delta SASsa^{1/2}$ (50)	0.442	0.704	0.543
	ΔDIs (20), $\Delta SASsa^{1/2}$ (50), Helix (88)	0.465	0.615	0.530
	Nphb (3), ΔDIs (20), $\Delta SASta^{1/2}$ (49)	0.419	0.692	0.522
4	Hydrophobicity (4), ΔDIs (20), $\Delta SASta^{1/2}$ (49), $esp3$ (83)	0.500	0.768	0.606
	Hydrophobicity (4), ΔDIs (20), $\Delta SASsa^{1/2}$ (50), $esp3$ (83)	0.488	0.737	0.587
	ΔDIs (20), $\Delta SASsa^{1/2}$ (50), $esp3$ (83), Helix (88)	0.512	0.667	0.579
5	Hydrophobicity (4), ΔDIs (20), SASbau (33), $\Delta SASta^{1/2}$ (49), $esp3$ (83)	0.570	0.754	0.649
	Nphb (3), ΔDIs (20), $\Delta SASsa^{1/2}$ (50), $esp3$ (83), Helix (88)	0.581	0.694	0.633
	Hydrophobicity (4), ΔDIs (20), $\Delta SASta^{1/2}$ (49), $\Delta SASnr^{1/2}$ (68), $esp3$ (83)	0.558	0.716	0.627
6	Nphb (3), PIsu (14), ΔDIs (20), $\Delta SASsa^{1/2}$ (50), $esp3$ (83), Helix (88)	0.616	0.726	0.667
	Nphb (3), PItu (13), ΔDIs (20), $\Delta SASsa^{1/2}$ (50), $esp3$ (83), Helix (88)	0.593	0.761	0.667
	Hydrophobicity (4), ΔDIs (20), ΔPIs (22), SASbau (33), $\Delta SASta^{1/2}$ (49), $esp3$ (83)	0.605	0.703	0.650
7	Nphb (3), PItu (13), ΔDIs (20), SAStau (29), $\Delta SASsa^{1/2}$ (50), $esp3$ (83), Helix (88)	0.628	0.750	0.684
	Nphb (3), PIsu (14), ΔDIs (20), SAStau (29), $\Delta SASsa^{1/2}$ (50), $esp3$ (83), Helix (88)	0.640	0.733	0.683
	Hydrophobicity (4), ΔDIs (20), ΔPIs (22), SAStau (29), SASbau (33), $\Delta SASta^{1/2}$ (49), $esp3$ (83)	0.640	0.705	0.671
8	Nphb (3), PIsu (14), ΔDIs (20), SAStau (29), $\Delta SASta^{\frac{1}{2}}$ (49), $\Delta SASsa^{1/2}$ (50), $esp3$ (83), Helix (88)	0.616	0.757	0.679
	Hydrophobicity (4), DIsb (16), ΔDIs (20), ΔPIs (22), SAStau (29), SASbau (33), $\Delta SASta^{1/2}$ (49), $esp3$ (83)	0.651	0.709	0.679
	Nphb (3), PItu (13), PIsu (14), ΔDIs (20), SAStau (29), $\Delta SASsa^{1/2}$ (50), $esp3$ (83), Helix (88)	0.651	0.700	0.675

[a] The number in the parenthesis is corresponding to the feature number in Table S5.

Table S8. The features selected and the corresponding cross validation performance in the SFS process based on the original 97 features.

Round	Features selected[a]	Recall	Precision	F1 score
1	SASnrb (46)	0.349	0.566	0.432
	relSAS (27)	0.302	0.650	0.413
	SASbrb (44)	0.314	0.600	0.412
2	ΔPIt (21), SASbrb (44)	0.407	0.700	0.515
	ΔPIt (21), relSAS (27)	0.395	0.708	0.507
	relSAS (27), $\Delta SASpa^2$ (63)	0.430	0.617	0.507
3	Nec (2), ΔPIt (21), SASbrb (44)	0.523	0.652	0.581
	ΔPIt (21), SASbrb (44), HB1 (86)	0.523	0.608	0.563
	ΔPIt (21), relSAS (27), SASsau (31)	0.500	0.642	0.562
4	Nec (2), PItb (17), ΔPIt (21), SASbrb (44)	0.593	0.638	0.614
	Nec (2), ΔPIt (21), SASbrb (44), $\Delta SASpr$ (71)	0.535	0.708	0.609
	Nec (2), ΔPIt (21), SASbrb (44), $\Delta SASna^2$ (64)	0.558	0.640	0.596

5	Nec (2), ΔPIt(21), SASbrb (44), ΔSASpr (71), CNSV_REL1_pss(96)	0.663	0.600	0.630
	Nec (2), ΔDIt (19), ΔPIt(21), SASbrb (44), $\Delta SASna^2$(64)	0.581	0.685	0.629
	Nec (2), PItb (17), PIsb(18), ΔPIt(21), SASbrb (44)	0.581	0.667	0.621
6	Nec (2), ΔDIt (19), ΔPIt (21), SASbrb (44), $\Delta SASna^2$ (64), $\Delta SAStr^{3/2}$ (73)	0.640	0.688	0.663
	Nec (2), ΔDIt (19), ΔPIt (21), SASbrb (44), $\Delta SASsa^{1/2}$ (50), $\Delta SASna^2$(64),	0.640	0.688	0.663
	Nec (2), ΔDIt (19), ΔPIt(21), SASsau (31), SASbrb (44), $\Delta SASna^2$(64)	0.640	0.679	0.659
7	Nec (2), ΔDIt (19), ΔPIt(21), SASbrb (44), $\Delta SASta^{1/2}$ (49), $\Delta SASsa^{1/2}$ (50), $\Delta SASna^2$(64)	0.651	0.683	0.667
	Nec (2), ΔDIt (19), ΔPIt(21), SASbrb (44), SASnab (45), $\Delta SASsa^{1/2}$ (50), $\Delta SASna^2$(64),	0.651	0.683	0.667
	Nec (2), ΔDIt (19), ΔPIt (21), SASbrb (44), $\Delta SASna^2$ (64), ΔSASsr(70), $\Delta SAStr^{3/2}$ (73)	0.651	0.675	0.663
8	Nec (2), ΔDIt (19), ΔPIt(21), SASbrb (44), $\Delta SASta^{1/2}$ (49), $\Delta SASsa^{1/2}$ (50), $\Delta SASna^{1/2}$(52), $\Delta SASna^2$(64)	0.663	0.687	0.675
	Nec (2), ΔDIt (19), ΔPIt(21), SASsab (41), SASbrb (44), $\Delta SASta^{1/2}$ (49), $\Delta SASsa^{1/2}$ (50), $\Delta SASna^2$(64)	0.663	0.687	0.675
	Nec (2), ΔDIt (19), ΔPIt (21), SASbrb (44), $\Delta SASna^2$ (64), ΔSASsr(70), $\Delta SAStr^{3/2}$ (73), esp4(84)	0.651	0.691	0.671
9	Nec (2), ΔDIt (19), ΔPIt(21), SASbrb (44), ΔSASpa (55) $\Delta SASna^2$(64), ΔSASsr(70), $\Delta SAStr^{3/2}$ (73), esp4(84)	0.663	0.722	0.691
	Nec (2), ΔDIt (19), ΔPIt(21), SASbru (34), SASbrb (44), $\Delta SASna^2$(64), ΔSASsr(70), $\Delta SAStr^{3/2}$ (73), esp4(84)	0.651	0.727	0.687
	Nec (2), ΔDIt (19), ΔPIt(21), SASbrb (44), $\Delta SASpa^{3/2}$(59), $\Delta SASna^2$(64), ΔSASsr(70), $\Delta SAStr^{3/2}$ (73), esp4(84)	0.651	0.718	0.683
10	Nec (2), ΔDIt (19), ΔPIt(21), SASbru (34), SASbrb (44), ΔSASpa (55) $\Delta SASna^2$(64), ΔSASsr(70), $\Delta SAStr^{3/2}$ (73), esp4(84)	0.686	0.766	0.724
	Nec (2), ΔDIt (19), ΔPIt(21), SASbru (34), SASbrb (44), $\Delta SASpa^{3/2}$(59), $\Delta SASna^2$(64), ΔSASsr(70), $\Delta SAStr^{3/2}$ (73), esp4(84)	0.663	0.750	0.704
	Nec (2), ΔDIt (19), ΔPIt(21), SASbru (34), SASbrb (44), SASprb (48), $\Delta SASna^2$(64), ΔSASsr(70), $\Delta SAStr^{3/2}$ (73), esp4(84)	0.663	0.750	0.704
11	Nec (2), ΔDIt (19), ΔPIt(21), SASbru (34), SASbrb (44), ΔSASpa (55) $\Delta SASna^2$(64), $\Delta SASsr^{1/2}$(66), ΔSASsr(70), $\Delta SAStr^{3/2}$ (73), esp4(84)	0.686	0.766	0.724
	Nec (2), Hdrpo (4), ΔDIt (19), ΔPIt (21), SASbru (34), SASbrb (44), $\Delta SASpa^{3/2}$(59), $\Delta SASna^2$(64), ΔSASsr(70), $\Delta SAStr^{3/2}$ (73), esp4(84)	0.698	0.741	0.719
	Nec (2), ΔDIt (19), ΔPIt(21), SASbru (34), SASbrb (44), SASprb (48), $\Delta SASna^2$(64), ΔSASsr(70), $\Delta SAStr^{3/2}$ (73), $\Delta SASsr^{3/2}$(74), esp4(84)	0.686	0.747	0.715

[a] The number in the parenthesis is corresponding to the feature number in Table S5.

Statistically analysis of the correlations between hotspots and different features

Residues' physicochemical characteristics

As shown in List 1 of the main text, three of the ten physicochemical features were selected by decision trees: Na (Number of atoms), Nphb (Number of potential hydrogen bonds) and Hdrpo (Hydrophobicity). We calculated the average values of these 3 features for hot spots and non-hot spots. As shown in Fig. S1, the average values of hot spot residues of two features (Na, Hdrpo) are larger than for non-hot spot residues. The differences of these 3 features between hot spots and non-hot spots were analyzed using the Wilcoxon Rank Sum test. As shown in Fig S1, the difference in values of Na is statistically significant with P-values of 0.0086, when comparing hot spots to non-hot spots. Among these 3 features, Nphb was selected in our final model by the sequential forward feature selection process. Although the difference of the feature between hot spots and non-hot spots are not statistically significant, they may be complementary to other selected features.

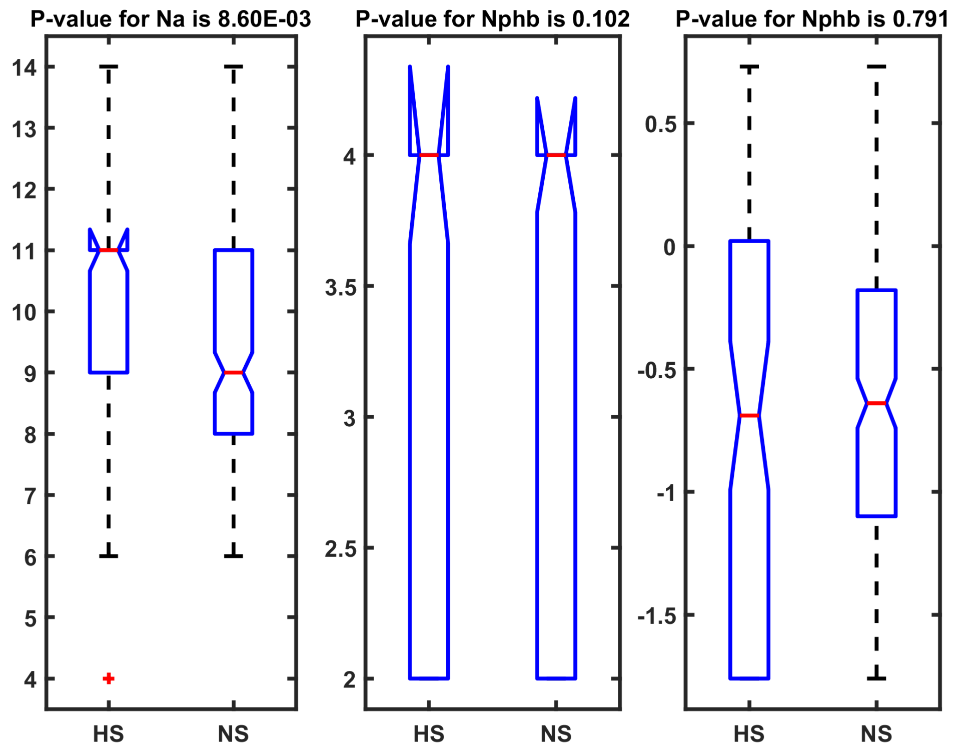

Fig. S1. Box-plots and P-values of the 3 physicochemical characteristics selected by decision tree.

Depth, protrusion index

As shown in List 1 of the main text, seven of the sixteen depth and protrusion index related features were selected by decision trees, which are PItu (average protrusion index of the total residue in unbound state), PIsu (average protrusion index of the residue side chain in unbound state), DItb (average depth index of the total residue in bound state), DIsb (average depth index of the side chain of the residue in bound state), ΔDIt (the difference of depth indexes (DI) of the total residue between bound and unbound state), ΔDIs (the difference of depth indexes (DI) of the side chain between bound and unbound state), and ΔPIs (the difference of protrusion indexes (PI) of the side chain between bound and unbound state). Obviously, the redundancy exists among the 7 selected features, as 4 of them are related to the depth index. We analyzed these 7 features selected by decision tree, as shown in Fig S2, the averages of ΔDIt and ΔDIs for hot spot residues are smaller than non-hot spot residues. On the contrary, the averages of PItu, PIsu, DItb, DIsb, and ΔPIs for hot spot residues are larger than non-hot spot residues. Based on Wilcoxon Rank Sum test, the differences of DItb, DIsb, ΔDIt, ΔDIs, and ΔPIs are statistically significant with p-values of 7.0E-04, 1.0E-04, 3.16E-08, 1.95E-08, and 6.90E-03, respectively.

After the SFS feature selection process, PItu and ΔDIs were kept in the final model. The average values of ΔDIs for hot spots is -1.02, which are statistically smaller than the average value for non-hot spots of -0.379. On the contrary, the average of PItu for hot spots is 0.972 that is larger than the average for non-hot spots of 0.846. However, the absolute average values of the two features for hot spot residues are all larger than the values of non-hot spot residues, which indicate the hot spot residues are buried deeper than non-hot spot residues.

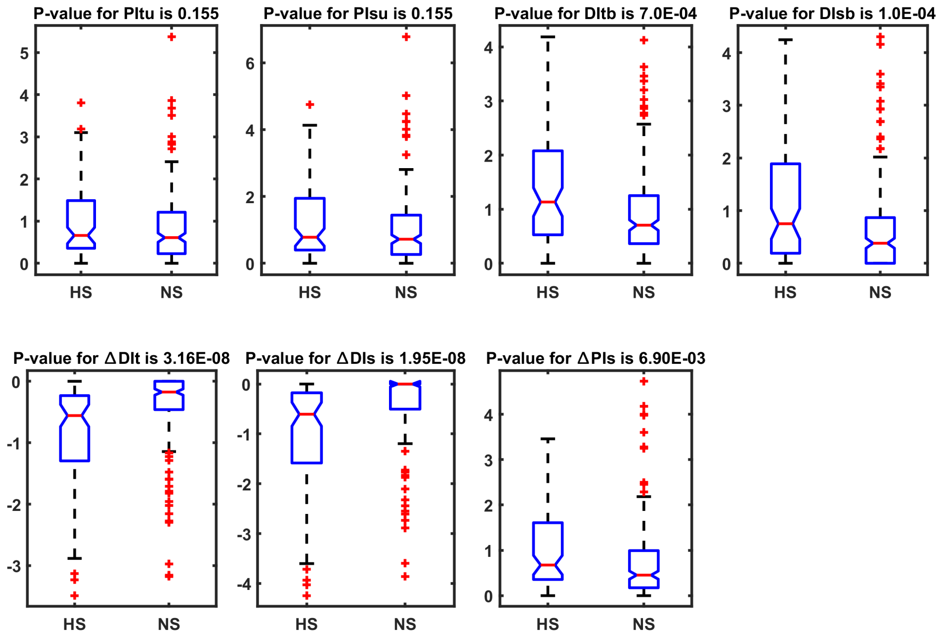

Fig. S2. Box-plots and P-values of the 7 depth, protrusion index related features selected by decision tree.

Solvent accessible surface area

As shown in List 1 of the main text, 6 of the 54 SASA related features were selected by decision trees, which are SAStau, SASbau, SASpau, $\Delta SASta^{1/2}$, $\Delta SASsa^{1/2}$, and $\Delta SASnr^{1/2}$. As shown in Fig. S3, the average values of the 5 features (SAStau, SASbau, $\Delta SASta^{1/2}$, $\Delta SASsa^{1/2}$, and $\Delta SASnr^{1/2}$) for hot spot residues are larger than for non-hot spot residues. By the Wilcoxon Rank Sum test, we analyzed the statistical significances of the differences of the 6 features for hot spot and non-hot spot residues. Fig S3 shows the p-values for $\Delta SASta^{1/2}$, $\Delta SASsa^{1/2}$and $\Delta SASnr^{1/2}$ are 3.20E-04, 2.57E-04 and 3.30E-03, which indicate the differences of these features are statistically significant.

After the SFS process, two of them were selected in the final model, which are SAStau and $\Delta SASsa^{1/2}$. Only the difference of $\Delta SASsa^{1/2}$ are statistically significant between hot spot and non-hot spot residues, which again implies the complementarity between different features. Noticed the feature $\Delta SASsa^{1/2}$ is a unique feature introduced only in this work and our previous work for predicting hot spot on protein-protein interfaces [1], which indicates the square root of the buried side chain solvent accessible surface areas may has more relationship to the binding affinity than the buried side chain solvent accessible surface area.

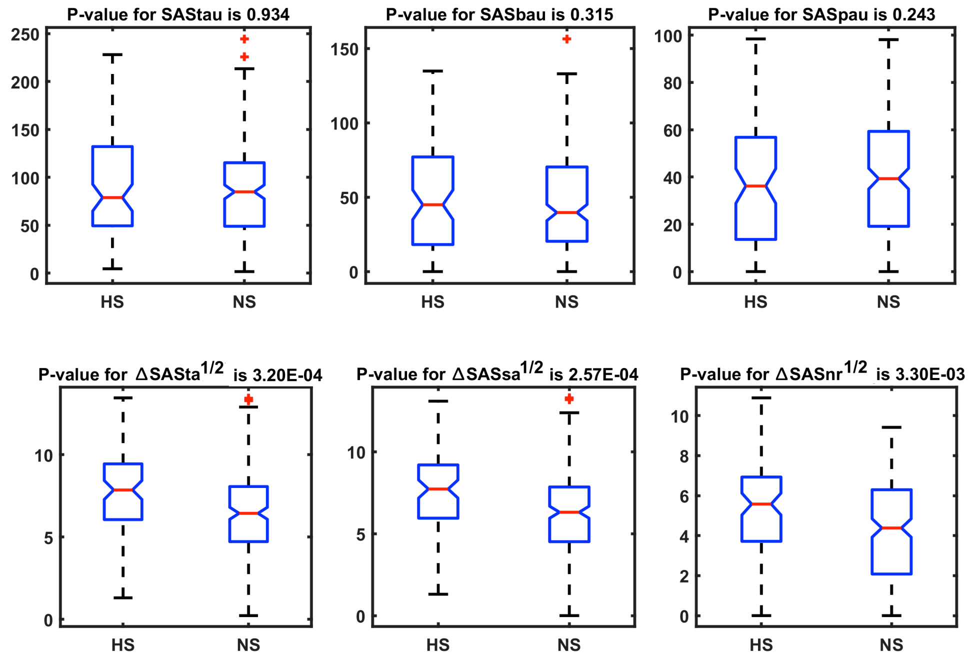

Fig. S3. Box-plots and P-values of the 6 SASA related features selected by decision tree.

Electrostatic potential

As shown in List 1 of the main text, 2 of the 5 electrostatic potential related features were selected by decision trees, which are esp1 and esp3. As shown in Fig. S4, the average values of esp1 and esp3 for hot spot residues are both larger than for non-hot spot residues. By the Wilcoxon Rank Sum test, we analyzed the statistical significances of the differences of the two features for hot spot and non-hot spot residues. Fig S4 shows the p-values for esp1 and esp3 are 4.30E-03 and 2.0E-03, which indicate the differences of these features are statistically significant.

After the SFS process, esp3 was selected in the final model. Esp3 is the electrostatic potential of the neighbor residues and the target residue, and esp1 is the electrostatic potential of the target residue. The p-value of esp3 is smaller than esp1, which indicate that the electrostatic potential of the residue patch is better than the single target residue in differentiating hot spot and non-hot spot residues. These electrostatic potential related features have been proposed to predict protein-DNA binding site in our previous works [2,

3], and the results in this work show that it is also an effective feature to predict hot spots within protein-NA interfaces.

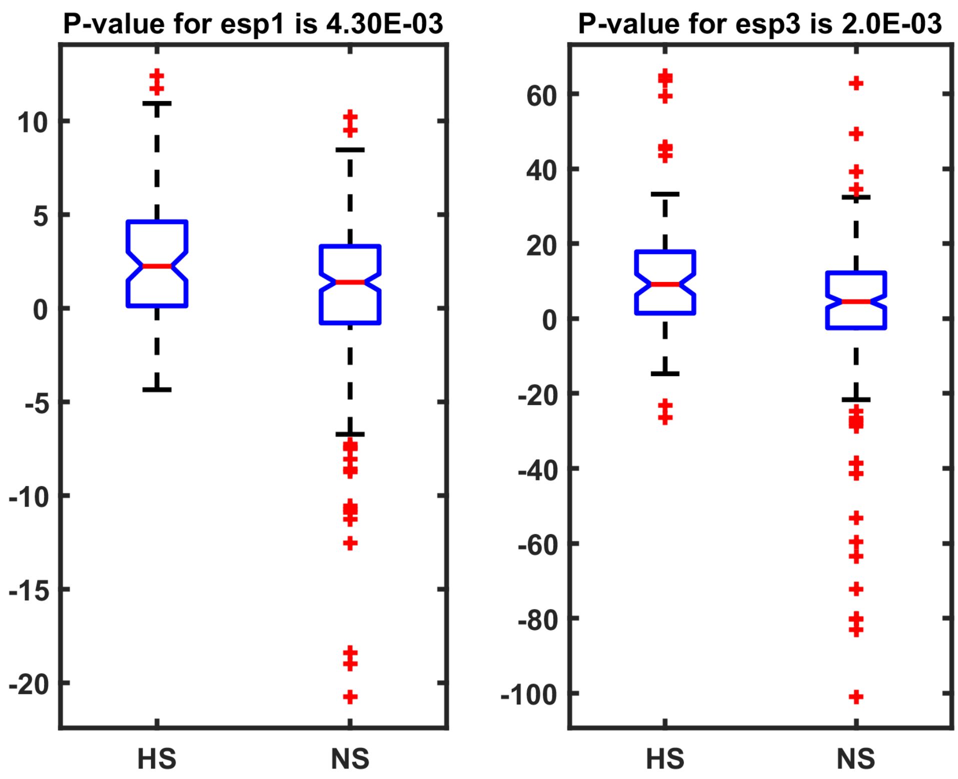

Fig. S4. Box-plots and P-values of the 2 electrostatic potential related features selected by decision tree.

Secondary structure

As shown in List 1 of the main text, only 1 of the 5 secondary structure related features were selected by decision trees, which is Helix. As shown in Fig S5, the average value of Helix of hot spot residues is 0.279 that is smaller than the value of non-hot spot residues, however, the difference is not statistically significant with a P-value of 0.408.

After the SFS process, the Helix was kept in the final model, which indicates that it may complement with other selected features.

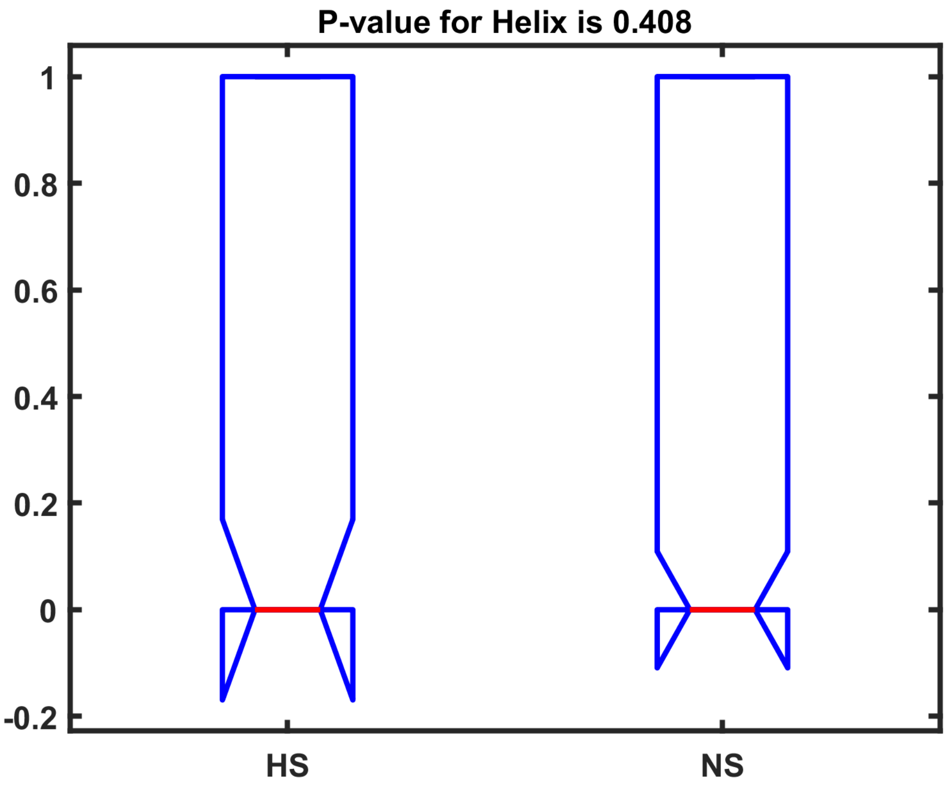

Fig. S5. Box-plots and P-value of the Helix feature by decision tree.

Conservation

In this study, we proposed 4 types new conservation related features in addition to the traditional conservation score based on information entropy. However, only the traditional conservation score, which was named as CNSV, was selected by the decision tree. As shown in Fig S6, the average CNSV of hot spot residues is 1.26 that is a little smaller than the value of non-hot spot residues 1.30, however, the difference is not statistically significant according to the P-value of 0.798.

After the SFS process, the CNSV was not selected in the final model.

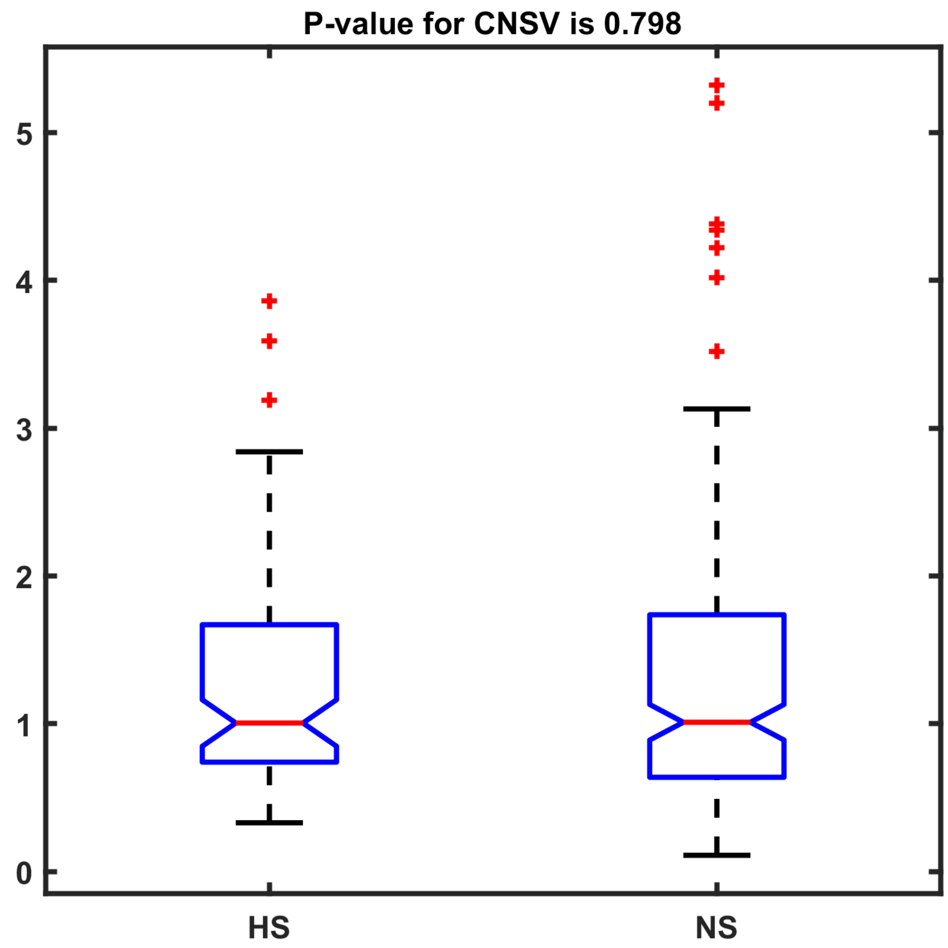

Fig. S6. Box-plots and P-values of the residue conservation features selected by decision tree.

References

1. Qiao Y, Xiong Y, Gao H, Zhu X, Chen P: **Protein-protein interface hot spots prediction based on a hybrid feature selection strategy**. *BMC bioinformatics* 2018, **19**(1):14.
2. Sukumar S, Zhu X, Ericksen SS, Mitchell JC: **DBSI server: DNA binding site identifier**. *Bioinformatics* 2016, **32**(18):2853-2855.
3. Zhu X, Ericksen SS, Mitchell JC: **DBSI: DNA-binding site identifier**. *Nucleic acids research* 2013, **41**(16):e160.
